# Fusion model combining ultrasound-based radiomics and deep transfer learning with clinical parameters for preoperative prediction of pelvic lymph node metastasis in cervical cancer

**DOI:** 10.3389/fonc.2025.1681029

**Published:** 2025-11-13

**Authors:** Jihan Wang, Shengxian Bao, Tongtong Huang, Yongzhi Cai, Binbin Jin, Ji Wu

**Affiliations:** 1 Department of Ultrasonic Medicine, the First Affiliated Hospital of Guangxi Medical University, Nanning, Guangxi, China; 2 Department of Ultrasonic Medicine, the Affiliated Tumor Hospital of Guangxi Medical University, Nanning, Guangxi, China

**Keywords:** cervical cancer, radiomics, lymph node metastasis, ultrasound, featurefusion, nomogram, deep transfer learning

## Abstract

**Background:**

To develop and validate a multimodal fusion model integrating ultrasound-based radiomics, deep transfer learning (DTL), and clinical parameters for preoperative pelvic lymph node metastasis (PLNM) prediction in cervical cancer.

**Methods:**

A retrospective cohort of 421 patients with surgically confirmed cervical cancer was divided into the training (70%, n = 294) and testing (30%, n = 127) sets. Ultrasound-based radiomics (1,561 handcrafted features) and 3 DTL architectures (DenseNet121, ResNet50, AlexNet) were employed for feature extraction. After redundancy reduction (Spearman correlation, least absolute shrinkage and selection operator regression) and principal component analysis, fused radiomics-DTL features were combined with clinical predictors. Eight machine learning classifiers were evaluated, and the optimal model was used to construct a nomogram. Performance was assessed using area under the curve (AUC), calibration curves, and decision curve analysis (DCA).

**Results:**

The multilayer perceptron-based fusion model achieved a testing AUC of 0.753, outperforming standalone radiomics (AUC = 0.729) and DTL models (best AUC = 0.702; DenseNet121). Integration of clinical predictors (maximum tumor diameter and red blood cell count) further enhanced performance, yielding a nomogram with training/testing AUCs of 0.871 and 0.764, and a testing sensitivity and specificity of 58.1% and 84.4%,respectively. DCA demonstrated superior clinical utility for the nomogram across threshold probabilities (10%–50%).

**Conclusions:**

We developed a multimodal fusion model integrating ultrasound-based radiomics, DTL, and clinical parameters for preoperative PLNM prediction in cervical cancer. The proposed nomogram provides a clinically applicable, cost-effective tool for preoperative PLNM prediction, particularly valuable for optimizing treatment decisions in resource-limited settings.

## Introduction

Cervical cancer is a critical global health challenge, as it is the fourth most prevalent malignancy among women worldwide (1). The clinical management of this disease hinges on precise staging according to the International Federation of Gynecology and Obstetrics (FIGO) criteria, particularly the assessment of pelvic lymph node metastasis (PLNM), which fundamentally alters the therapeutic pathway (2, 3). Current National Comprehensive Cancer Network (NCCN) guidelines recommend radical hysterectomy with pelvic lymphadenectomy for stage IB–IIA lesions (4), yet emerging evidence questions this approach given that 70%–90% of patients with early-stage cervical cancer derive no oncological benefit from nodal dissection while facing substantial complication risks (2, 5). The 2018 revision of the FIGO criteria emphasized this prognostic stratification by introducing stage IIIC for radiologically suspected nodal involvement, and mandating chemoradiation over surgical intervention for these lesions (6). This staging evolution heightens the need for the accurate preoperative assessment of PLNM status, as treatment algorithms become increasingly dependent on imaging findings rather than surgical pathology (7).The reliable preoperative prediction of PLNM could significantly alter clinical management, potentially sparing node-positive patients from primary surgery and its associated morbidity, and instead directing them towards definitive chemoradiation, as supported by clinical evidence (8).

Unfortunately, current diagnostic modalities exhibit critical limitations in nodal evaluation, and the low incidence of intraoperatively confirmed nodal metastases (3.9%) despite preoperative imaging evaluation (9). Compared to CT, magnetic resonance imaging (MRI) demonstrates improved performance through functional sequences, yet a meta-analysis has revealed persistent interobserver variability exceeding 20% in lymph node characterization (10). Positron-emission tomography (PET)/CT, though valuable for metabolic assessment, suffers from limited accessibility and high false-negative rates in sub-centimeter nodes (11). Ultrasonography, the most widely available modality, shows operator-dependent accuracy, with its sensitivity differing according to the operator’s experience (12). Sentinel lymph node biopsy is the most precise technique for assessing PLNM preoperatively; however, this approach is invasive, and its outcomes may be affected by factors such as atypical lymphatic drainage patterns, the effectiveness of preoperative lymphoscintigraphy, and the level of surgical expertise (13, 14). These diagnostic challenges have catalyzed innovation in quantitative imaging analysis.

Radiomics enables the high-throughput extraction of subvisual tumor features through the mathematical characterization of texture heterogeneity, margin irregularity, and vascular patterns (15), while deep learning (DL) techniques, especially convolutional neural network architectures, can identify intricate patterns and characteristics linked to lymph node metastasis (LNM). These advanced computational approaches significantly improve the precision of LNM prediction models (16). Although radiomic characteristics and DL-derived features each possess unique strengths and inherent constraints, the synergistic combination of these methodologies provides mutually reinforcing diagnostic insights. Consequently, this integrative approach has emerged as a significant focus area in contemporary medical imaging research (17). The multimodal fusion of handcrafted radiomic features and deep transfer learning (DTL)-derived biomarkers has improved diagnostic accuracy beyond single-modality approaches in breast cancers (18).

The biological rationale for the utility of ultrasonography in nodal assessment resides in its unique capacity to characterize dynamic tumor-stromal interactions through real-time functional imaging. Shear-wave elastography(SWE) provides biomechanical insights by measuring alterations in tissue stiffness caused by metastatic desmoplastic reactions, which are a critical discriminator between reactive and malignant lymph nodes (19). This multifaceted capability for biological profiling positions ultrasonography as the ideal platform for developing predictive models that bridge radiological findings with underlying metastatic pathophysiology (20).

The clinical imperative for non-invasive nodal assessment extends beyond diagnostic accuracy. Unnecessary lymphadenectomy contributes to chronic lymphedema and increased healthcare costs (21, 22). In contrast, ultrasound-based predictive models offer cost-effective solutions adaptable to diverse healthcare settings. This methodology specifically addresses the limitations of prior MRI-centric radiomic models that require specialized sequences (such as diffusion-weighted imaging and apparent diffusion coefficient mapping), which are unavailable in low-resource regions. Currently, there is limited literature on the application of integrated models combining ultrasound radiomics and deep transfer learning for predicting pelvic lymph node metastasis in cervical cancer.

The primary objective of this investigation is to establish a clinically translatable decision-support tool that enables the non-invasive stratification of pelvic nodal metastasis risk in cervical cancer patients. Specifically, we aim to accomplish the following (1): validate ultrasound-derived radiomic signatures against surgical pathology in a 421-patient cohort (2); determine the complementary value of DTL-derived features in augmenting the value of the ultrasound-based radiomic signatures; and (3) develop an interpretable predictive model integrating quantitative imaging biomarkers with routine clinical and hematological parameters. This initiative directly responds to the guidelines of the European Society of Gynecological Oncology, European Society for Radiotherapy and Oncology, and European Society of Pathology, which advocate for precision-medicine approaches in gynecological oncology, while addressing healthcare disparities through ultrasound-centric technology, which is accessible across resource settings (23, 24).

## Methods

### Cohort selection

**Figure 1:**
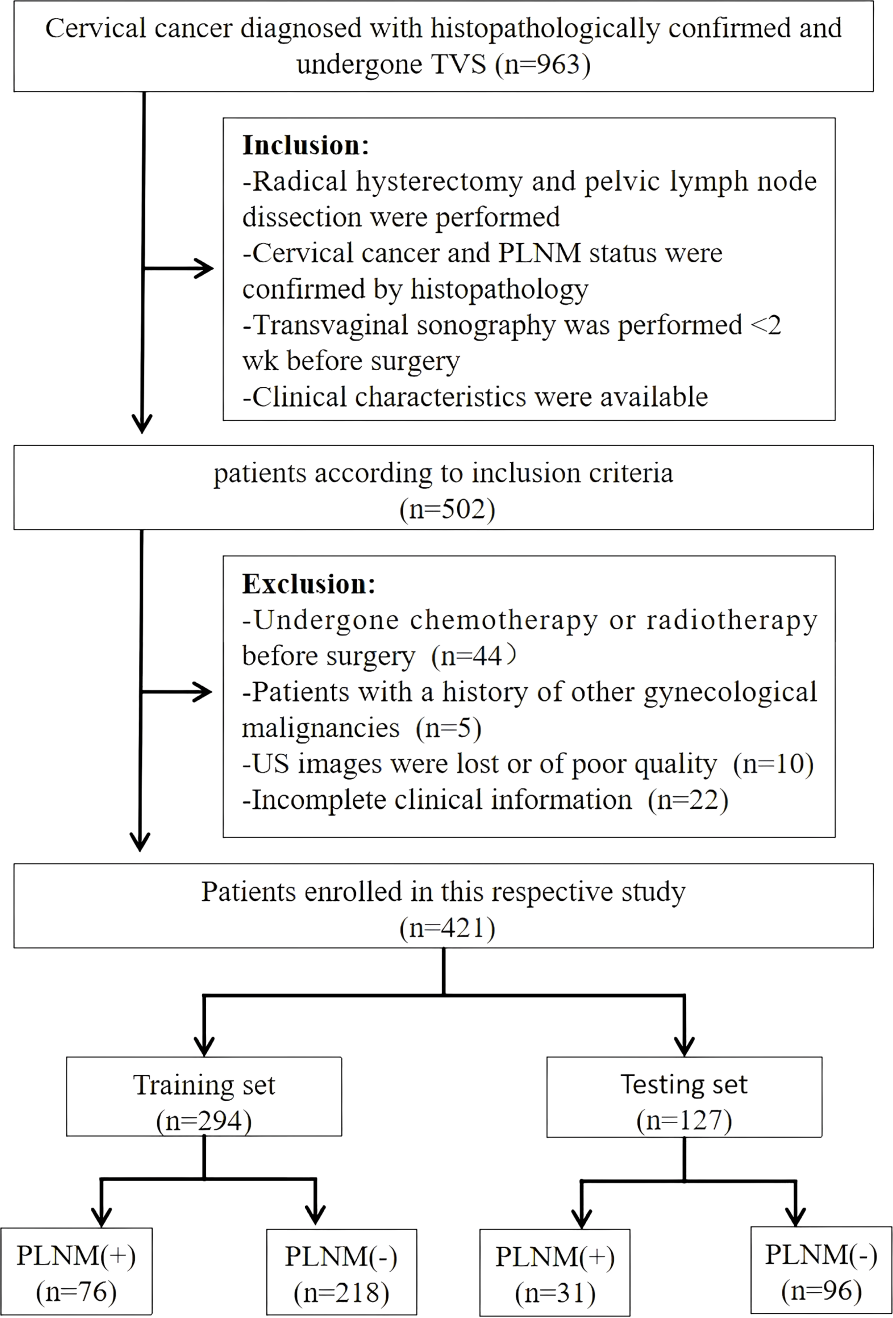
Flowchart of patient enrollment, exclusion, and grouping. TVS, transvaginal ultrasonography; PLNM, pelvic lymph node metastasis; US, ultrasound.

Patients from the First Affiliated Hospital of Guangxi Medical University undergoing radical hysterectomy with pelvic lymphadenectomy between May 2020 and September 2024 were screened against predefined eligibility criteria. Inclusion required the following (1): histopathologically confirmed cervical cancer with definitive PLNM status (2), preoperative transvaginal ultrasound imaging within 2 weeks before surgery capturing measurable lesions, and (3) availability of complete clinical records. Individuals meeting any of the following criteria were excluded from the study: prior oncological treatments (chemotherapy/radiotherapy), other concurrent gynecological malignancies, non-diagnostic ultrasound images, or incomplete data. From an initial pool of 963 candidates, 421 patients met the selection criteria and were enrolled in the study (**Figure 1**).

### Clinical and imaging data acquisition

**Figure 2:**
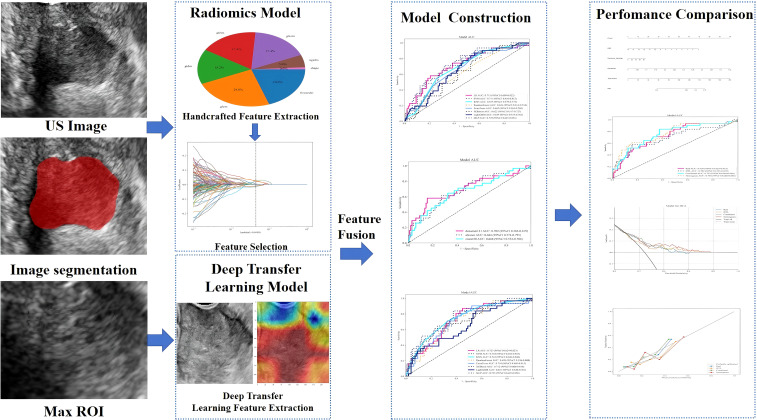
Schematic workflow of the study design. The framework includes image acquisition, feature extraction (radiomics and deep transfer learning), feature fusion, model construction, and nomogram development. US, ultrasonography; ROI, region of interest.

We systematically extracted the following demographic, hematological, and histopathological parameters from the patients’ electronic health records: age, parity, abortion history, body mass index (BMI), hematological indices (red blood cell [RBC] count, white blood cell [WBC] count, eosinophil count, and alkaline phosphatase [ALP]), levels of tumor markers (cancer antigen 125 [CA125], CA153, CA199, carcinoembryonic antigen [CEA], and squamous cell carcinoma [SCC] antigen), maximum lesion diameter, histological type, and PLNM status according to the postoperative pathological results (see the study design on **Figure 2**).

Ultrasound examinations were performed using the GE Voluson E10 and Logiq E9 systems equipped with 5–9 MHz transvaginal transducers. Standardized imaging protocols mandated bladder evacuation 10 min before the procedure, lithotomy positioning, and the acquisition of 6–10 representative images per patient in orthogonal planes. A single maximally cross-sectional image per patient was archived in the hospital’s picture archiving system for blinded analysis by 2 sonographers with 5 and 11 years of experience. Discrepancies in lesion characterization were resolved through consensus review.

### Tumor segmentation and reproducibility assessment

Manual delineation of tumor boundaries was performed using ITK-SNAP (*v*3.8.0) on the selected ultrasound slices. To assess inter-observer variability, we implemented a dual-annotation protocol: Sonographer A segmented all images, while Sonographer B independently annotated 50 randomly selected images. Intra-observer variability was assessed through repeated segmentations by Sonographer A after an 8-week interval. Features exhibiting intraclass correlation coefficients(ICCs) < 0.75 in both the inter- and intra-observer analyses were excluded to ensure robustness.

### Radiomic feature extraction and selection

Quantitative radiomic profiling extracted 1,561 handcrafted features via PyRadiomics (*v*3.0.1), categorized into the following 3 domains: geometric morphology (42 features), intensity distributions (744 features), and textural heterogeneity (775 features). Textural characterization employed 4 matrix-based methods: gray-level co-occurrence, gray-level run-length (GLRLM), gray-level size-zone, and neighboring gray tone difference matrices. A triphasic feature-reduction pipeline was applied (1): Mann-Whitney *U* tests (*p* < 0.05) identified metastasis-associated features (2); Spearman correlation filtering (|ρ| > 0.9) eliminated redundant variables; and (3) least absolute shrinkage and selection operator (LASSO) regression with 10-fold cross-validation (λ = 0.0450, minimum criteria) selected 9 non-collinear predictors.

### Deep transfer learning architecture and optimization

Three convolutional neural networks DenseNet121, ResNet50, and AlexNet were adapted using transfer learning from ImageNet pretrained weights. Input images underwent region of interest (ROI)-centered cropping to exclude extralesional tissue, followed by Z-score normalization. To enhance the generalizability of the images, we performed real-time data augmentation via random cropping (± 15% volume), horizontal/vertical flipping (*p* = 0.5), and intensity scaling (± 20%). Feature embeddings were extracted from the penultimate fully connected layer (16,383 dimensions) of the optimal-performing network and reduced to 64 principal components via principal component analysis for computational efficiency.

### Multimodal feature fusion strategy

Hybrid biomarkers were developed by concatenating the radiomic signatures with the DTL-derived principal components, thereby generating a 73-dimensional feature space. Recursive feature elimination was then performed, prioritizing variables that demonstrated complementary predictive value. After this step, 7 radiomic and 9 DTL components were retained. The fused feature set was standardized (using z-scores) prior to model integration.

### Predictive modeling framework

Eight machine learning classifiers were evaluated: logistic regression(LR; L2 regularization), support vector machines (SVM; radial basis kernel), k-nearest neighbors (KNN; k = 5), RandomForest (100 trees), ExtraTrees (50 trees), XGBoost (max_depth = 6), LightGBM (num_leaves = 31), and multilayer perceptron (MLP; 2 hidden layers, ReLU activation). Hyperparameter optimization employed a grid search with 10-fold cross-validation on the training cohort (70%, n = 294), prioritizing balanced accuracy. The MLP architecture incorporated dropout regularization (rate = 0.3) and early stopping (patience = 10 epochs) to mitigate overfitting.

### Clinical-radiomic nomogram development

Multivariate logistic regression identified independent clinical predictors (maximum tumor diameter and RBC count), which were integrated with the optimal fusion model outputs to construct a nomogram. Calibration slopes were adjusted using Platt scaling to align the predicted probabilities with the observed metastasis rates.

### Statistical validation protocol

Model discrimination was quantified using area under the receiver operating characteristic curve (AUC). Calibration accuracy was assessed using the Hosmer-Lemeshow test and Brier scores. Decision curve analysis evaluated clinical utility across probability thresholds (0%–100%). Continuous variables were analyzed using the Mann-Whitney *U* test or Student *t*-test following Shapiro-Wilk normality testing, while categorical variables were evaluated using the chi-square or Fisher exact test. Correlation analyses were performed using Pearson (normal distribution) or Spearman (non-parametric) coefficients.

### Computational infrastructure

All analyses were implemented using Python *v*3.9 (scikit-learn *v*1.2, PyTorch *v*1.13) on an NVIDIA A100 GPU cluster. Reproducibility was ensured through fixed random seeds (NumPy = 42, PyTorch = 3407) and version-controlled pipelines.

## Results

### Clinical and ultrasound characteristics​

This retrospective analysis included 421 cervical cancer patients, of whom 294 patients were allocated to the training cohort (218 [74.1%] PLNM-negative patients; 76 [25.9%] PLNM-positive patients) and 127 to the testing cohort (96 [75.6%] PLNM-negative patients; 31 [24.4%] PLNM-positive patients; **Table 1**). The PLNM-negative and PLNM-positive groups significantly differed in terms of parturition (*p* = 0.037), WBC count (*p* = 0.027), ALP level (*p* = 0.003), CA125 level (*p* < 0.001), CEA level (*p* = 0.002), SCC antigen level (*p* < 0.001), and maximum tumor diameter (*p* < 0.001). No significant differences were detected in age, abortion history, BMI, RBC count, and histology. Cohort stratification ensured balanced clinical characteristics between the training and testing sets (*p* > 0.05 for all variables).

**Table 1:** Demographic and ultrasonographic characteristics of the study participants.

Feature	Training (N = 294)	PLNM (–)	PLNM (+)	*P* value	Testing (N = 127)	PLNM (–)	PLNM (+)	*P* value
Age	52.93 ± 10.68	52.78 ± 10.69	53.38 ± 10.72	0.671	51.98 ± 10.17	51.15 ± 10.22	54.55 ± 9.72	0.091
Parturition	2.46 ± 1.32	2.39 ± 1.28	2.64 ± 1.42	0.309	2.52 ± 1.48	2.40 ± 1.51	2.90 ± 1.35	0.037
Abortion	1.41 ± 1.38	1.41 ± 1.39	1.43 ± 1.38	0.767	1.30 ± 1.32	1.34 ± 1.40	1.16 ± 1.07	0.726
BMI	23.35 ± 3.53	23.29 ± 3.43	23.52 ± 3.82	0.596	23.29 ± 3.39	23.47 ± 3.54	22.75 ± 2.83	0.594
RBC count (10^^12^/L)	4.09 ± 0.91	4.12 ± 0.99	3.99 ± 0.59	0.500	4.05 ± 0.62	4.09 ± 0.56	3.90 ± 0.78	0.138
WBC count (10^^9^/L)	11.14 ± 3.62	11.31 ± 3.65	10.64 ± 3.52	0.163	11.26 ± 3.86	11.66 ± 3.98	10.01 ± 3.22	0.027
ALP (U/L)	63.08 ± 19.26	61.86 ± 19.22	66.59 ± 19.06	0.015	65.13 ± 22.48	61.36 ± 18.33	76.81 ± 29.53	0.003
Eosinophil count (10^^9^/L)	0.16 ± 1.23	0.18 ± 1.42	0.10 ± 0.22	<0.001	0.05 ± 0.10	0.04 ± 0.10	0.06 ± 0.09	0.122
CA125 (U/mL)	37.53 ± 117.05	26.16 ± 49.86	70.13 ± 211.85	<0.001	34.93 ± 73.63	25.58 ± 61.65	63.91 ± 97.91	<0.001
CA153 (U/mL)	14.31 ± 8.95	13.66 ± 9.03	16.15 ± 8.48	0.008	13.27 ± 7.33	12.69 ± 6.80	15.04 ± 8.67	0.21
CA199 (U/mL)	46.76 ± 390.57	13.23 ± 24.10	142.92 ± 762.65	<0.001	21.87 ± 86.47	25.11 ± 99.00	11.82 ± 15.04	0.31
CEA (U/mL)	6.04 ± 16.58	5.08 ± 10.85	8.80 ± 26.89	0.024	4.14 ± 6.14	3.43 ± 5.10	6.34 ± 8.33	0.002
SCC antigen (ng/mL)	6.17 ± 9.99	5.00 ± 8.47	9.53 ± 12.92	0.002	6.91 ± 12.64	3.87 ± 5.90	16.32 ± 20.96	<0.001
Maximum diameter (mm)	33.10 ± 13.55	30.25 ± 11.86	41.29 ± 14.81	<0.001	31.83 ± 11.84	29.47 ± 10.91	39.16 ± 11.77	<0.001
Histology				0.167				0.075
Squamous cell carcinoma	231 (78.57)	174 (79.82)	57 (75.00)		101 (79.53)	72 (75.00)	29 (93.55)	
Adenocarcinoma	48 (16.33)	36 (16.51)	12 (15.79)		19 (14.96)	18 (18.75)	1 (3.23)	
Others	15 (5.10)	8 (3.67)	7 (9.21)		7 (5.51)	6 (6.25)	1 (3.23)	

PLNM, pelvic lymph node metastasis; BMI, body mass index; RBC, red blood cell; WBC, white blood cell; ALP, alkaline phosphatase; CA, cancer antigen; CEA, carcinoembryonic antigen; SCC antigen, squamous cell carcinoma antigen.

Univariate logistic regression identified age, parturition, abortion, BMI, RBC count, WBC count, ALP level, CA153 level, histology, and maximum tumor diameter as potential predictors of PLNM status (**Table 2**). Subsequent multivariate analysis confirmed maximum tumor diameter (odds ratio [OR] = 1.055, 95% confidence interval [CI]: 1.036–1.075) and RBC count (OR = 0.646, 95% CI: 0.459–0.910) as independent risk factors for PLNM.

**Table 2:** Univariate and multivariate logistic regression analysis of clinical predictors for PLNM.

Clinical and US characteristics	Univariate analysis				Multivariate analysis			
	OR	Lower 95% CI	Upper 95% CI	*P*	OR	Lower 95% CI	Upper 95% CI	*P*
Age	0.981	0.977	0.985	0.000	0.994	0.971	1.017	0.667
Parturition	0.730	0.672	0.792	0.000	1.155	0.951	1.404	0.222
Abortion	0.667	0.591	0.745	0.000	1.085	0.909	1.294	0.446
BMI	0.957	0.948	0.966	0.000	0.936	0.881	0.995	0.073
RBC count	0.771	0.731	0.815	0.000	0.646	0.459	0.910	0.036
WBC count	0.911	0.894	0.930	0.000	0.965	0.904	1.031	0.379
ALP	0.986	0.982	0.989	0.000	1.006	0.993	1.020	0.460
Eosinophil count	0.628	0.211	1.865	0.482				
CA125	1.000	0.998	1.001	0.860				
CA153	0.952	0.939	0.966	0.000	1.010	0.985	1.036	0.507
CA199	1.001	1.000	1.002	0.363				
CEA	0.988	0.974	1.003	0.182				
SCC antigen	0.981	0.964	0.999	0.077				
Maximum tumor diameter	0.981	0.974	0.986	0.000	1.055	1.036	1.075	0.000
Histology	0.602	0.427	0.848	0.015	1.410	0.911	2.181	0.196

PLNM, pelvic lymph node metastasis; US, ultrasonography; OR, odds ratio; CI, confidence interval; BMI, body mass index; RBC, red blood cell; WBC, white blood cell; ALP, alkaline phosphatase; CA, cancer antigen; CEA, carcinoembryonic antigen; SCC antigen, squamous cell carcinoma antigen.

### Radiomics model development​

**Figure 3:**
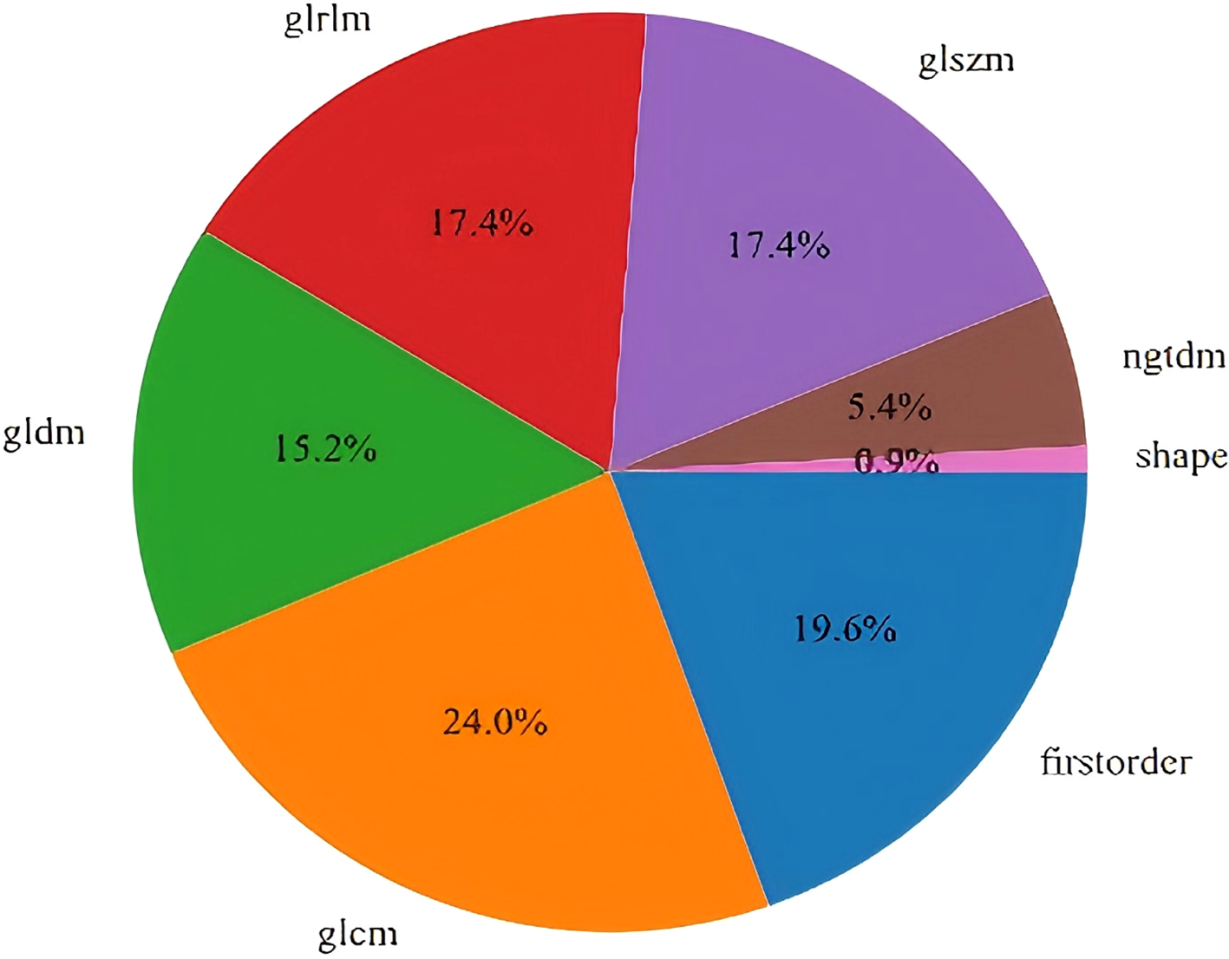
Distribution of 1,561 handcrafted radiomic features across 3 categories: geometric, intensity-based, and texture-based characteristics.

**Figure 4:**
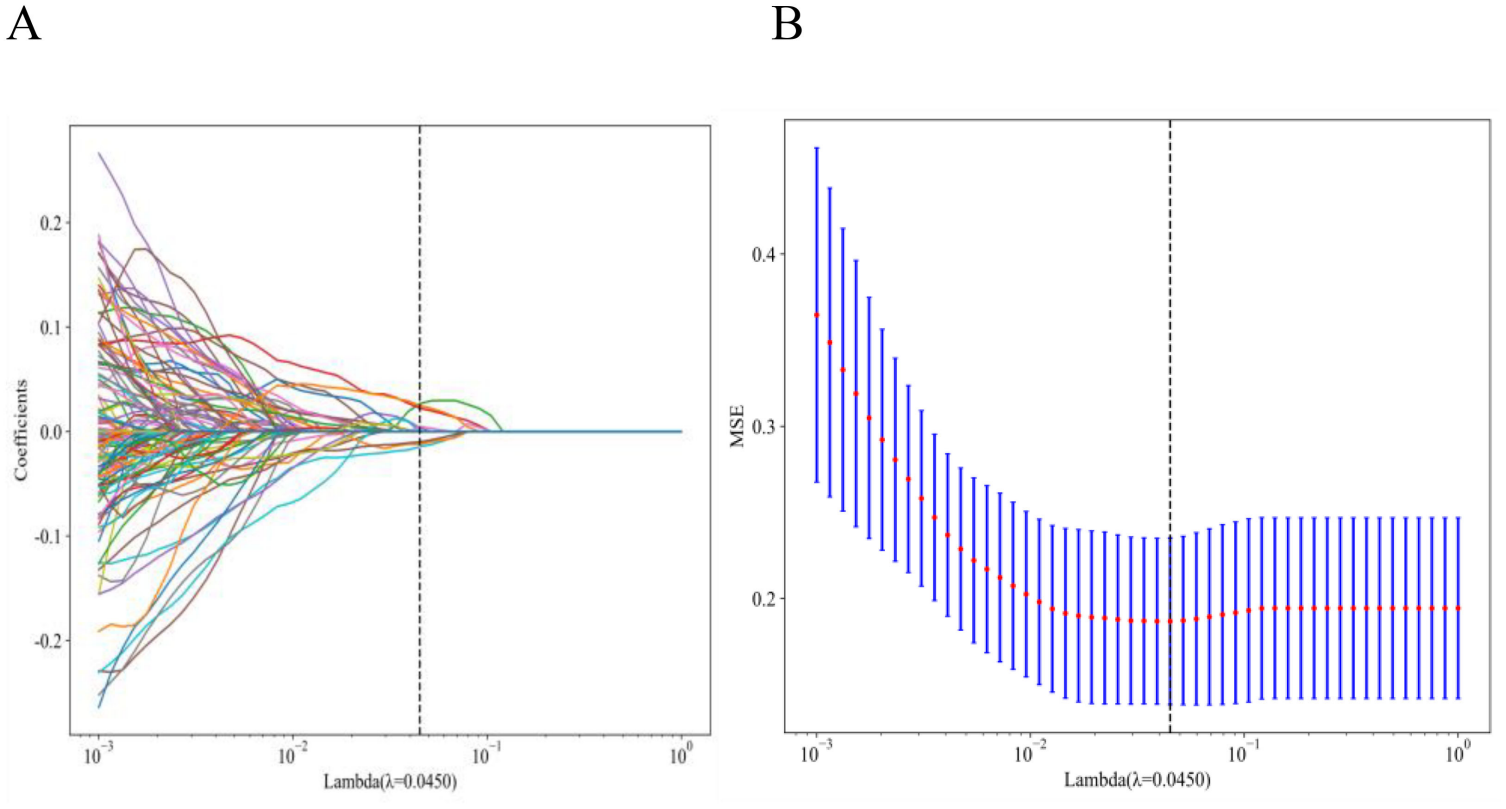
Handcrafted radiomic feature selection using LASSO **(A)** and histogram of the importance scores of selected radiomic features **(B)**. The optimal λ value was 0.0450. LASSO, least absolute shrinkage and selection operator.

**Figure 5:**
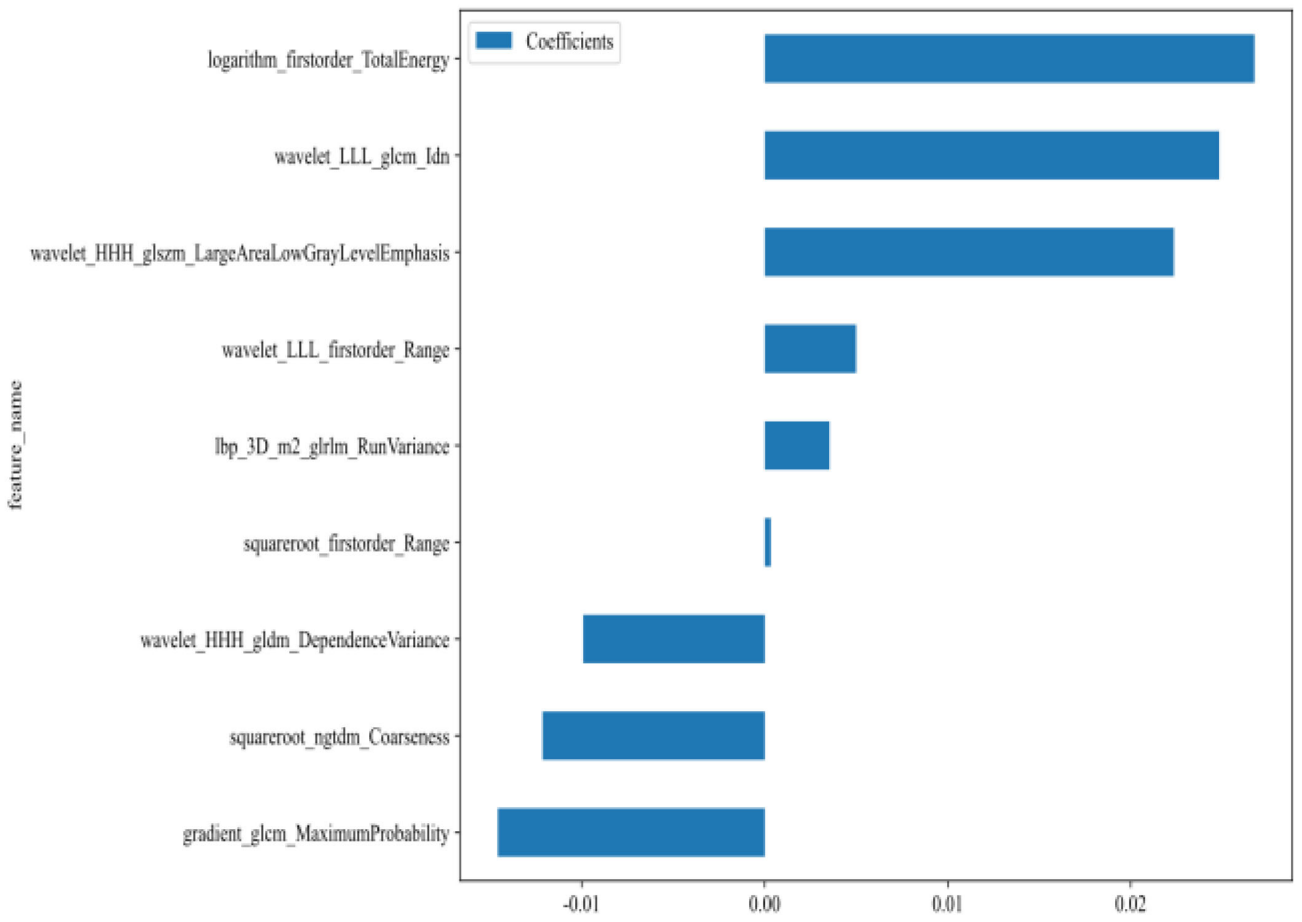
Histogram of the Rad-scores of selected features.

A total of 1,561 handcrafted radiomic features were extracted from the ultrasound images, and distributed across the geometric (n = 42), intensity-based (n = 744), and texture-based (n = 775) categories (**Figure 3**). Feature selection involved redundancy elimination via Spearman correlation (|ρ| > 0.8) followed by LASSO regression (λ = 0.0450, 10-fold cross-validation), and yielded 9 robust handcrafted radiomic features (**Figures 4**, **5**). The importance scores of these features, ranked by absolute LASSO coefficients, are visualized in **Figure 4B**.

**Figure 6:**
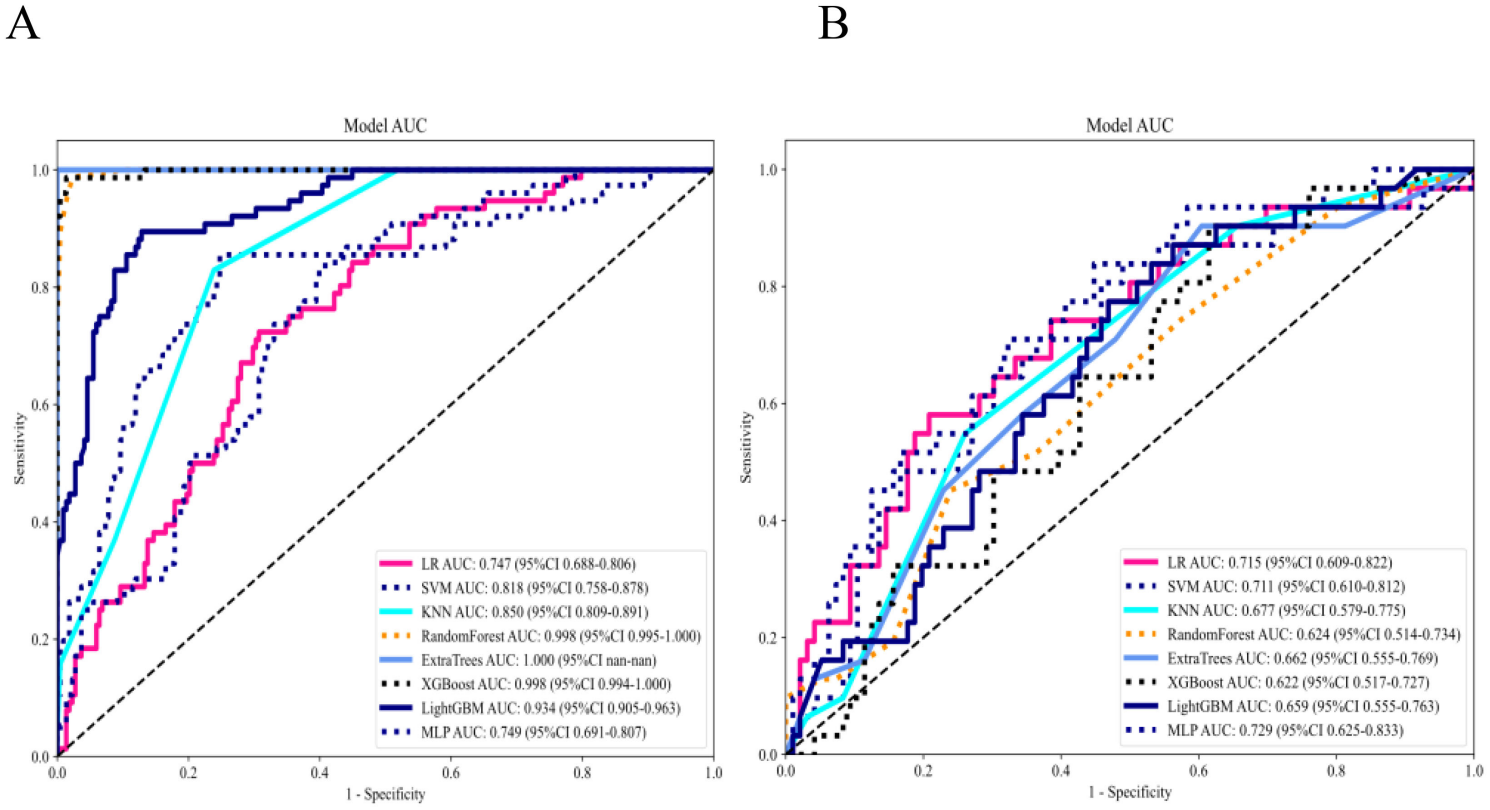
**(A)** Receiver operating characteristic (ROC) curves of 8 machine learning classifiers for the radiomics model in the training set. **(B)** ROC curves of 8 machine learning classifiers for the radiomics model in the testing set.

Eight machine-learning classifiers were evaluated using the selected handcrafted radiomic features (**Table 3**). The MLP model demonstrated optimal performance in the testing cohort (AUC = 0.729, 95% CI: 0.6249–0.8334), outperforming the logistic regression (AUC = 0.715), support vector machine (AUC = 0.711), and other classifiers (**Figure 6**).

**Table 3:** Diagnostic performance of radiomic models using different classifiers.

Model	AUC	95% CI	Accuracy	Sensitivity	Specificity	PPV	NPV
LR-Training	0.747	0.6877–0.8059	0.697	0.711	0.693	0.446	0.873
SVM-Training	0.818	0.7583–0.8781	0.776	0.842	0.752	0.542	0.932
KNN-Training	0.850	0.8093–0.8914	0.772	0.368	0.913	0.596	0.806
RandomForest-Training	0.998	0.9950–1.0000	0.976	0.947	0.986	0.960	0.982
ExtraTrees-Training	1.000	1.0000–1.0000	0.741	0.000	1.000	0.000	0.741
XGBoost-Training	0.998	0.9941–1.0000	0.983	0.974	0.986	0.961	0.991
LightGBM-Training	0.934	0.9051–0.9633	0.874	0.882	0.872	0.705	0.955
MLP-Training	0.749	0.6906–0.8071	0.653	0.829	0.592	0.414	0.908
LR-Testing	0.715	0.6086–0.8222	0.732	0.548	0.792	0.459	0.844
SVM-Testing	0.711	0.6095–0.8118	0.614	0.806	0.552	0.368	0.898
KNN-Testing	0.677	0.5785–0.7750	0.717	0.097	0.917	0.273	0.759
RandomForest-Testing	0.624	0.5141–0.7342	0.685	0.194	0.844	0.286	0.764
ExtraTrees-Testing	0.662	0.5553–0.7693	0.567	0.710	0.521	0.324	0.847
XGBoost-Testing	0.622	0.5167–0.7266	0.504	0.871	0.385	0.314	0.902
LightGBM-Testing	0.659	0.5554–0.7632	0.535	0.839	0.437	0.325	0.894
MLP-Testing	0.729	0.6249–0.8334	0.677	0.677	0.677	0.404	0.867

AUC, area under the curve; CI, confidence interval; PPV, positive predictive value; NPV, negative predictive value; LR, logistic regression; SVM, support vector machine; KNN, k-nearest neighbor; XGBoost, eXtreme gradient boost; LightGBM, light gradient boosting machine; MLP, multilayer perceptron.

### Deep transfer learning model performance

**Figure 7:**
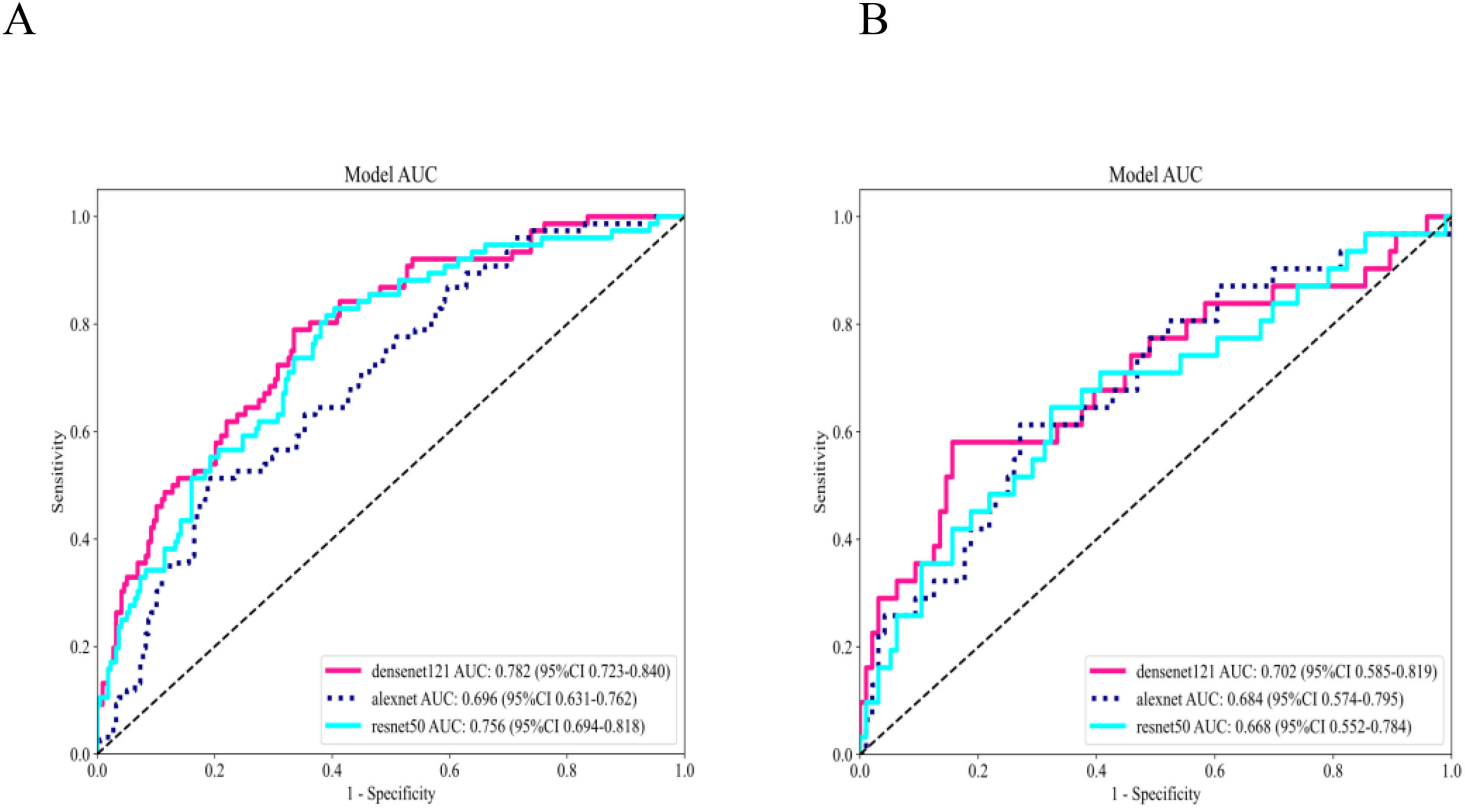
Performance evaluation of 3 DTL models in the training set **(A)** and testing set **(B)**. DenseNet121 outperformed ResNet50 and AlexNet, yielding an AUC of 0.702 in the testing set. DTL, deep transfer learning; AUC, area under the curve.

**Figure 8:**
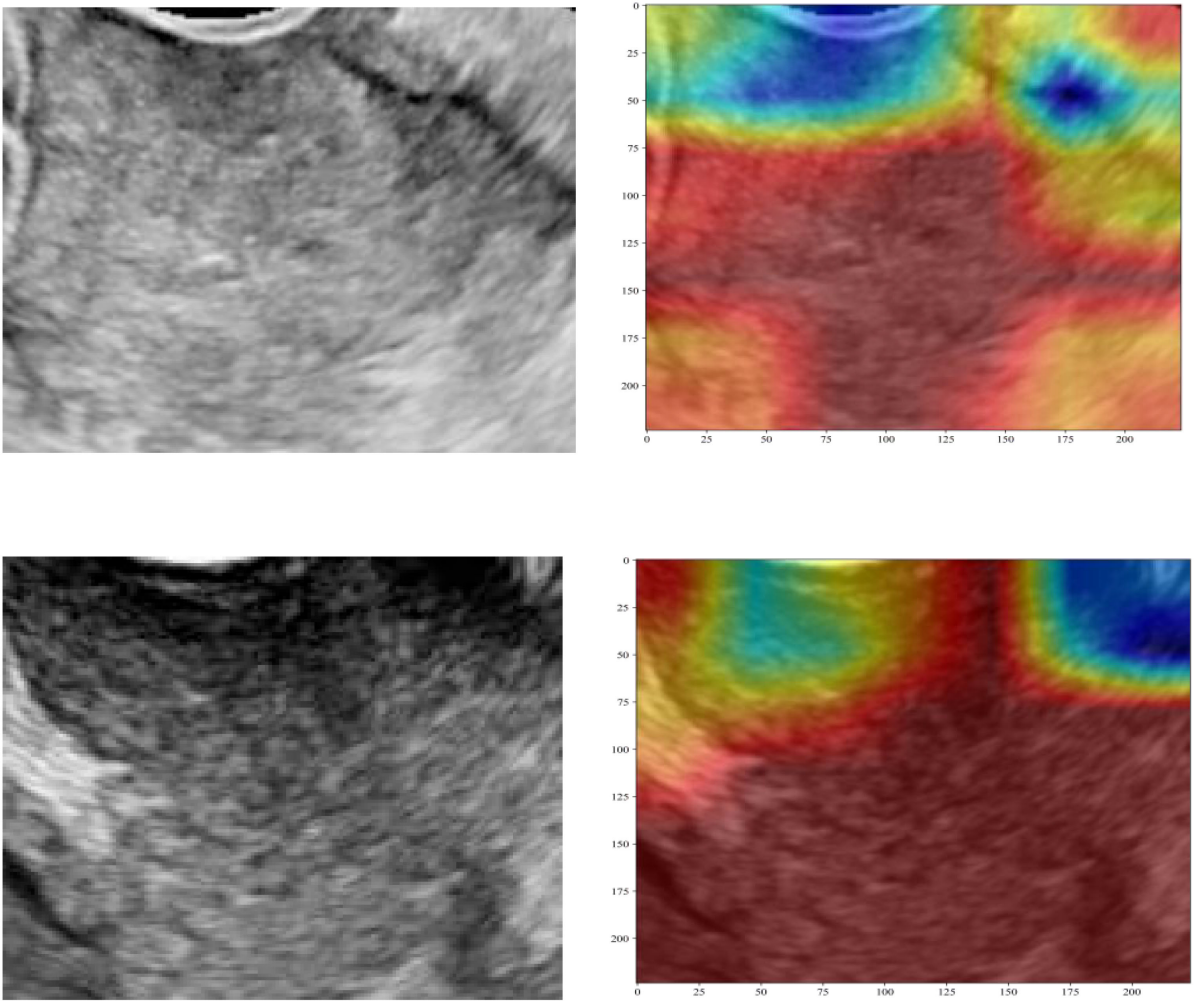
Gradient-weighted Class Activation Mapping visualization of the predictions of DenseNet121. The highlighted regions indicate tumor areas critical for PLNM classification. PLNM, pelvic lymph node metastasis.

Three pre-trained convolutional neural networks (DenseNet121, ResNet50, and AlexNet) were adapted for DTL analysis (**Table 4**). DenseNet121 achieved the highest testing AUC of 0.702 (95% CI: 0.5853–0.8193), surpassing ResNet50 (AUC = 0.668) and AlexNet (AUC = 0.684; **Figure 7**). Gradient-weighted Class Activation Mapping was used to localize the tumor subregions that were critical for the predictions made by DenseNet121, and the results revealed preferential attention to areas of heterogeneous echogenicity (**Figure 8**).

**Table 4:** Comparison of deep transfer learning models for PLNM prediction.

Model	AUC	95% CI	Accuracy	Sensitivity	Specificity	PPV	NPV
DenseNet121-Training	0.782	0.7231–0.8400	0.694	0.776	0.665	0.447	0.895
AlexNet-Training	0.696	0.6306–0.7622	0.728	0.500	0.807	0.475	0.822
ResNet50-Training	0.756	0.6939–0.8179	0.660	0.803	0.610	0.418	0.899
DenseNet121-Testing	0.702	0.5853–0.8193	0.772	0.548	0.844	0.531	0.853
AlexNet-Testing	0.684	0.5742–0.7947	0.693	0.581	0.729	0.409	0.843
ResNet50-Testing	0.668	0.5516–0.7837	0.661	0.613	0.677	0.380	0.844

PLNM, pelvic lymph node metastasis; AUC, area under the curve; CI, confidence interval; PPV, positive predictive value; NPV, negative predictive value.

### Feature fusion and combined model

**Figure 9:**
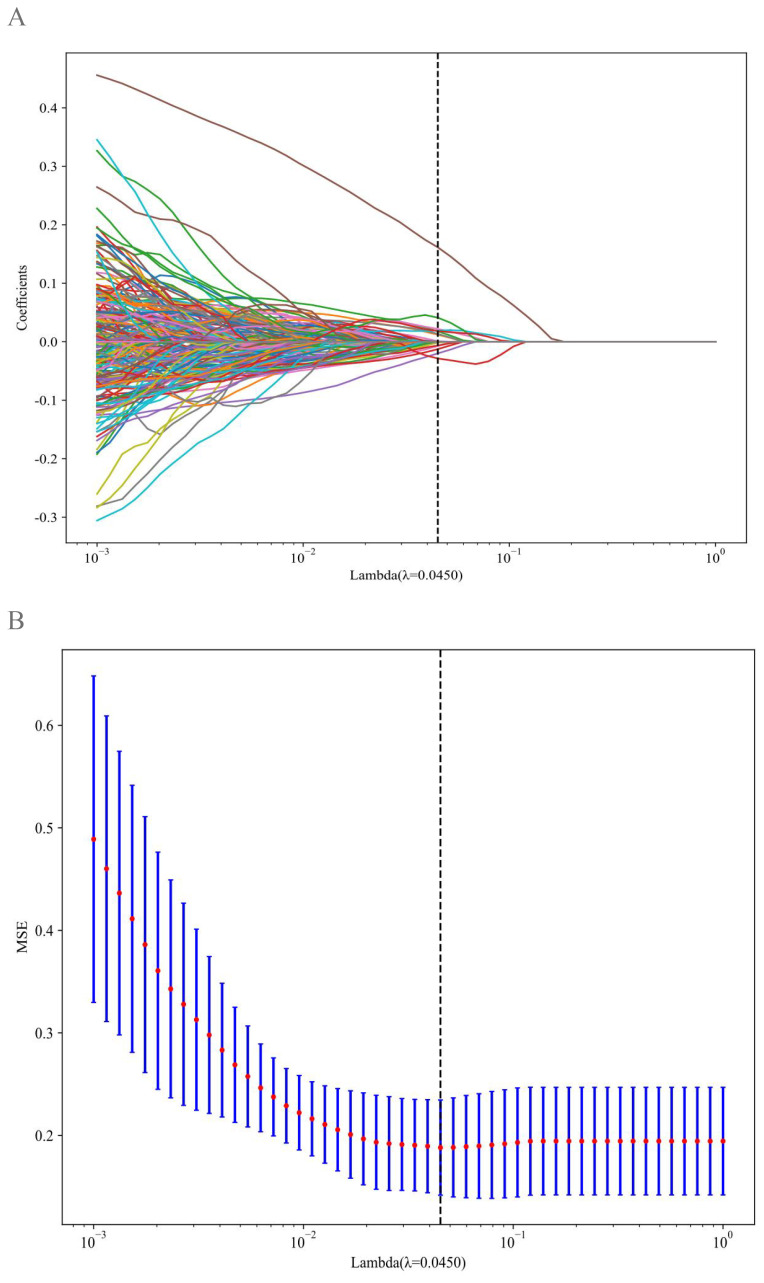
Fusion feature selection using LASSO **(A)** and histogram of the importance scores of the selected features **(B)**. The optimal l value was 0.0450. LASSO, least absolute shrinkage and selection operator.

**Figure 10:**
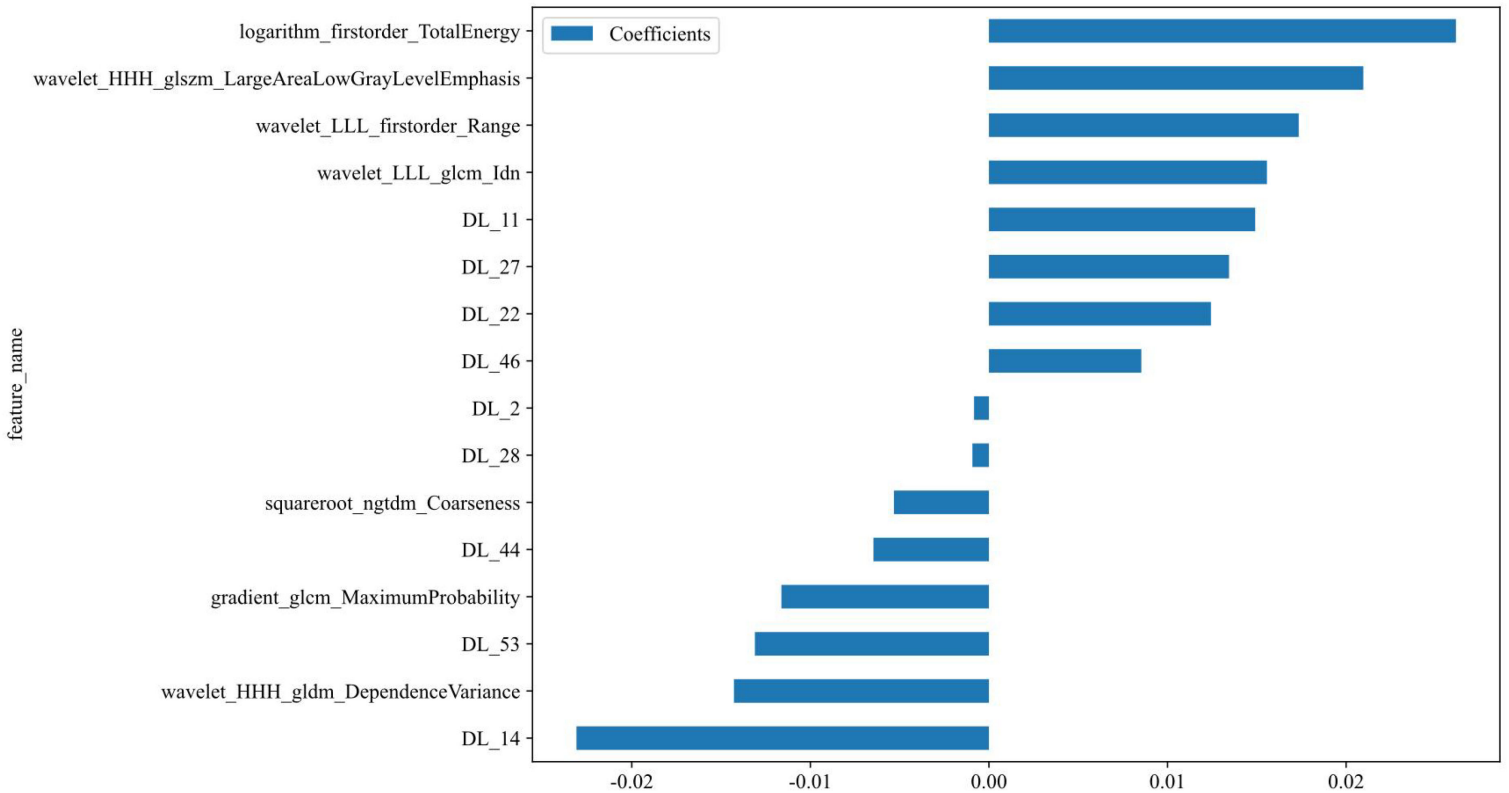
Selected fusion features and corresponding coefficients.

**Figure 11:**
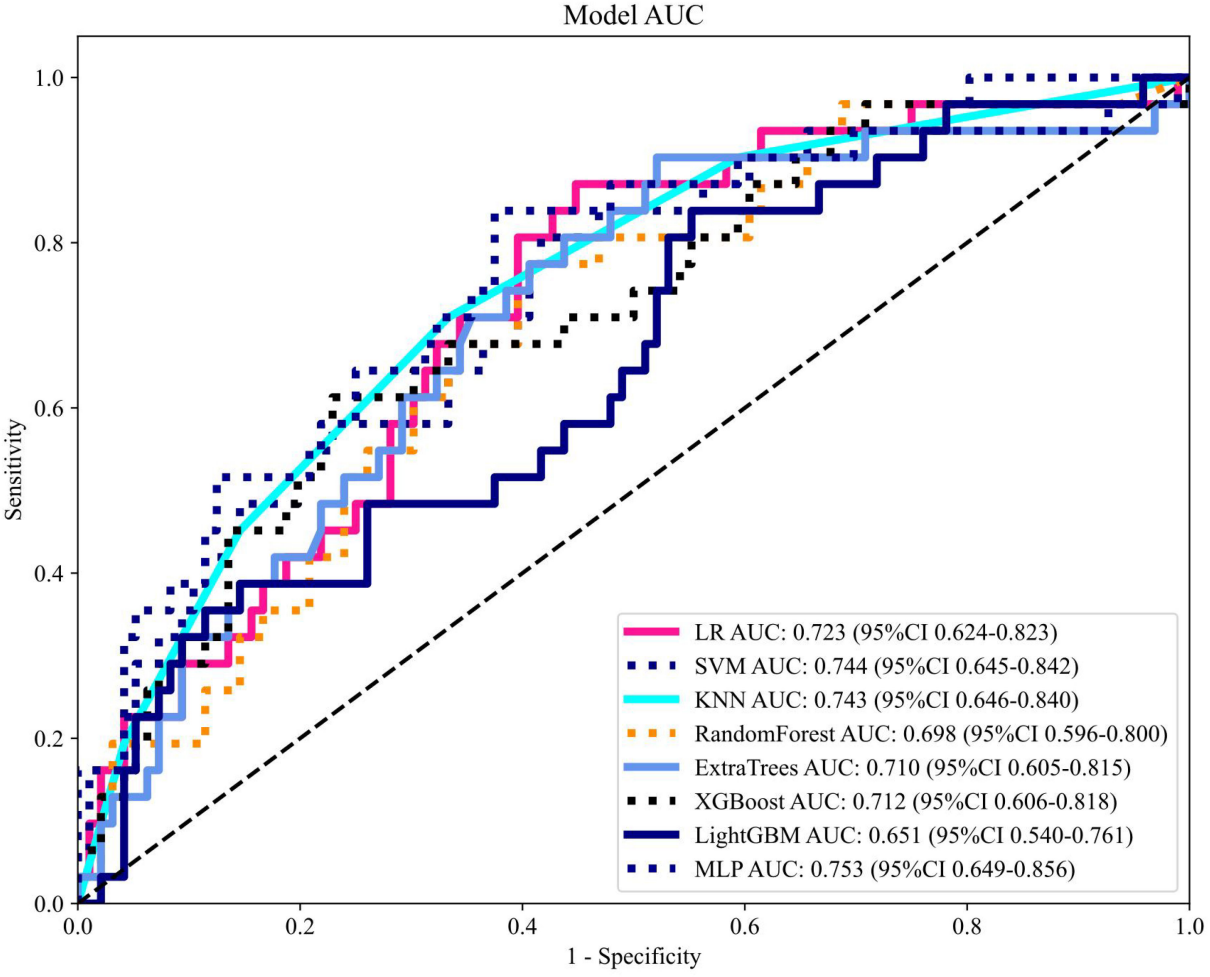
Receiver operating characteristic curve analysis of fused feature models. The MLP-based combined model achieved superior performance (AUC: 0.753) in the testing cohort. MLP, multilayer perceptron; AUC, area under the curve.

After recursive feature elimination, a total of 7 handcrafted radiomic features and 9 DTL features were retained from the fused feature set comprising 1625 dimensions (**Figures 9**, **10**). Among the 8 classifiers evaluated (**Table 5**), the MLP-based fusion model achieved a testing AUC of 0.753 (95% CI: 0.6494–0.8560), representing a 5.1% improvement over standalone DenseNet121 (AUC: 0.753 *vs*.0.702; **Figure 11**).

**Table 5:** Performance evaluation of feature fusion models across classifiers.

Model	AUC	95% CI	Accuracy	Sensitivity	Specificity	PPV	NPV
LR-Training	0.831	0.7768–0.8858	0.820	0.658	0.876	0.649	0.880
SVM-Training	0.922	0.8810–0.9627	0.857	0.921	0.835	0.660	0.968
KNN-Training	0.861	0.8179–0.9031	0.820	0.513	0.927	0.709	0.845
RandomForest-Training	0.869	0.8204–0.9176	0.776	0.816	0.761	0.544	0.922
ExtraTrees-Training	0.828	0.7727–0.8833	0.728	0.816	0.697	0.484	0.916
XGBoost-Training	0.975	0.9580–0.9924	0.935	0.895	0.950	0.861	0.963
LightGBM-Training	0.937	0.9051–0.9693	0.878	0.882	0.876	0.713	0.955
MLP-Training	0.857	0.8086–0.9064	0.762	0.829	0.739	0.525	0.925
LR-Testing	0.723	0.6243–0.8226	0.622	0.839	0.552	0.377	0.914
SVM-Testing	0.744	0.6451–0.8422	0.630	0.774	0.583	0.375	0.889
KNN-Testing	0.743	0.6451–0.8422	0.756	0.452	0.854	0.500	0.828
RandomForest-Testing	0.698	0.5956–0.8003	0.630	0.742	0.594	0.371	0.877
ExtraTrees-Testing	0.710	0.6053–0.8154	0.575	0.871	0.479	0.351	0.920
XGBoost-Testing	0.712	0.6058–0.8176	0.724	0.581	0.771	0.450	0.851
LightGBM-Testing	0.651	0.5399–0.7612	0.535	0.806	0.448	0.321	0.878
MLP-Testing	0.753	0.6494–0.8560	0.669	0.806	0.625	0.410	0.909

AUC, area under the curve; CI, confidence interval; PPV, positive predictive value; NPV, negative predictive value; LR, logistic regression; SVM, support vector machine; KNN, k-nearest neighbor; XGBoost, eXtreme gradient boost; LightGBM, light gradient boosting machine; MLP, multilayer perceptron.

### Nomogram construction and validation​

**Figure 12:**
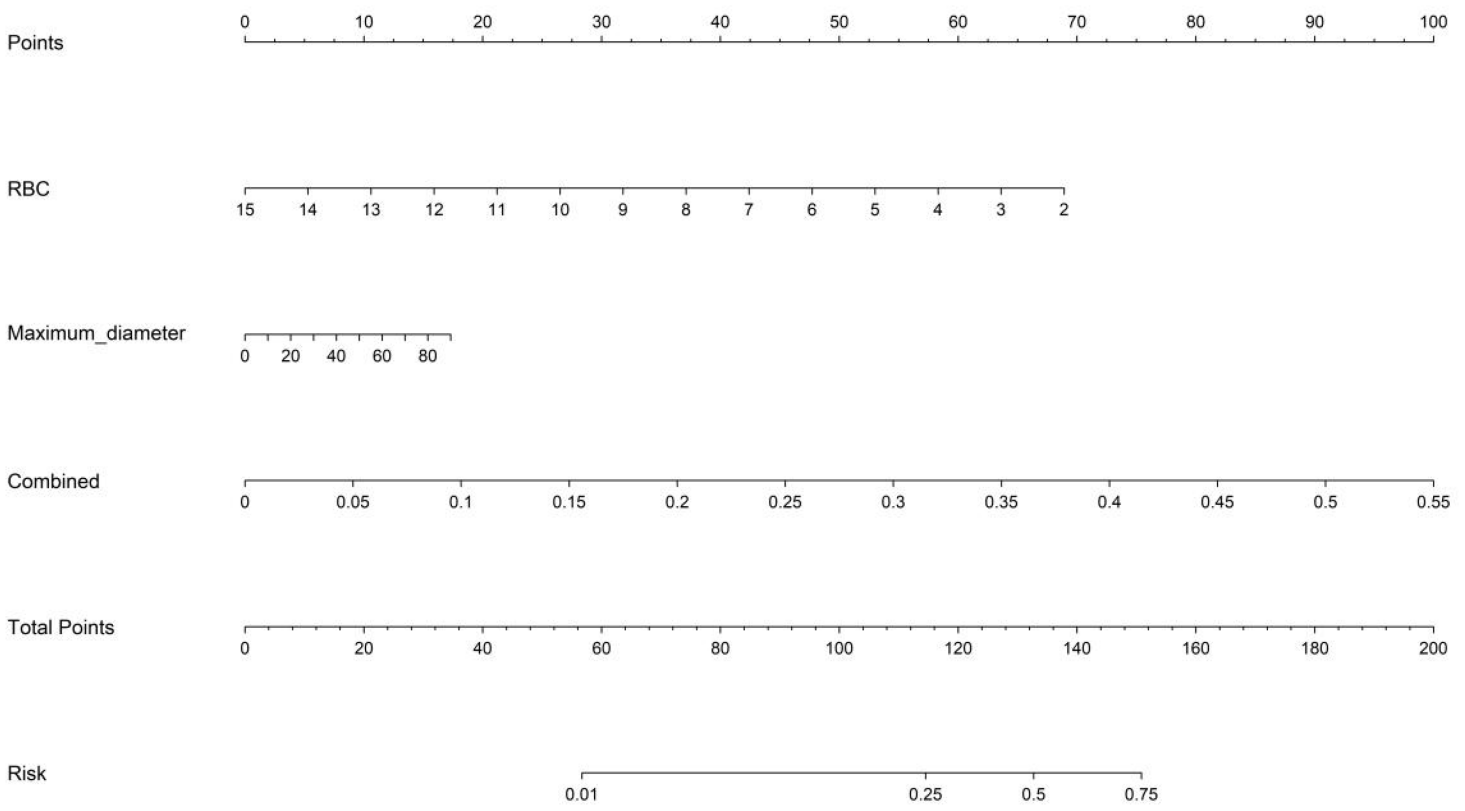
Nomogram incorporating fused radiomic-DTL features, maximum tumor diameter, and RBC count for individualized PLNM risk prediction. DTL, deep transfer learning; RBC, red blood cell; PLNM, pelvic lymph node metastasis.

**Figure 13:**
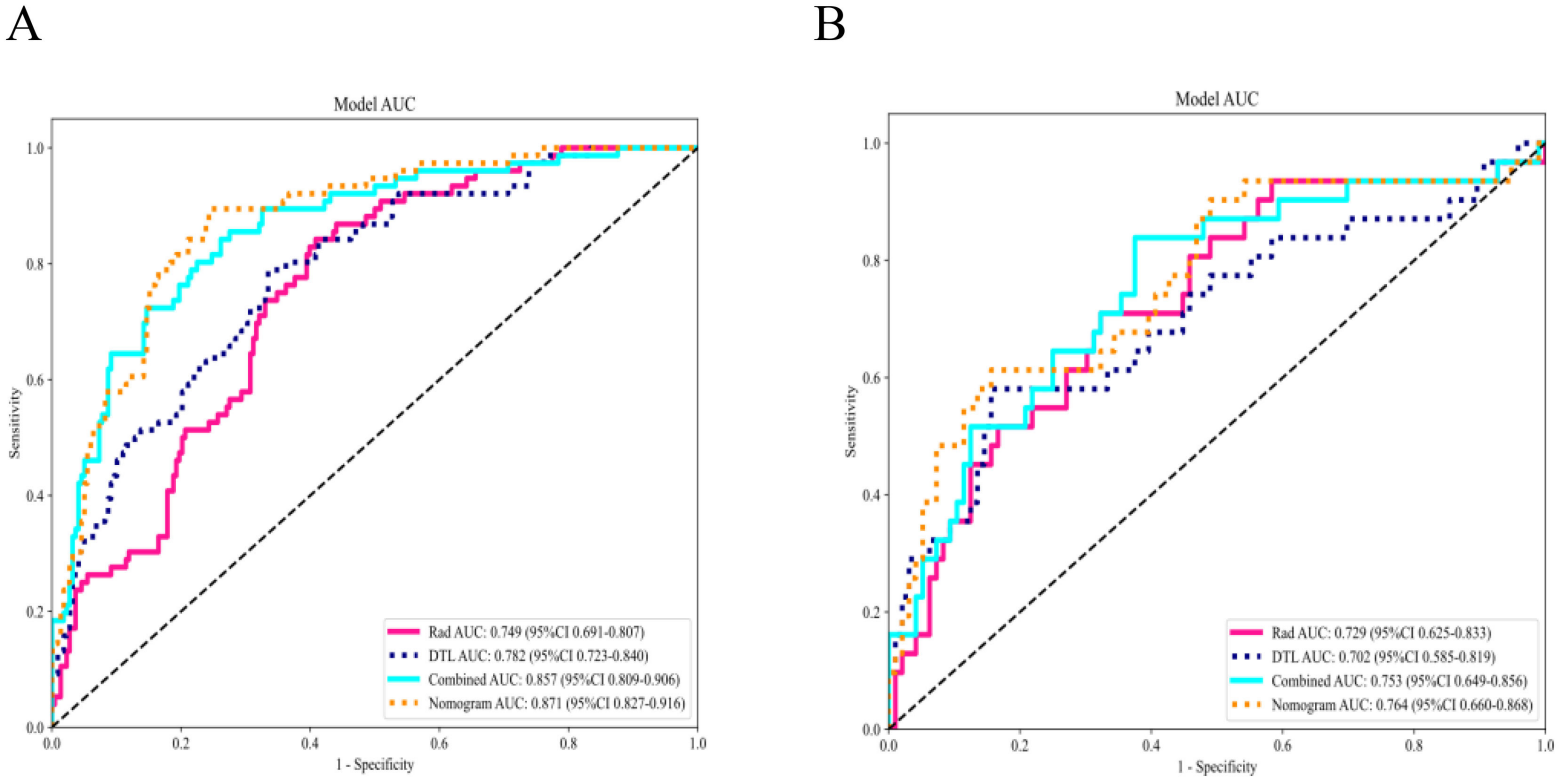
Comparison of receiver operating characteristic curves of all models. The nomogram demonstrated optimal diagnostic accuracy in both the training **(A)** and testing cohorts **(B)**.

**Figure 14:**
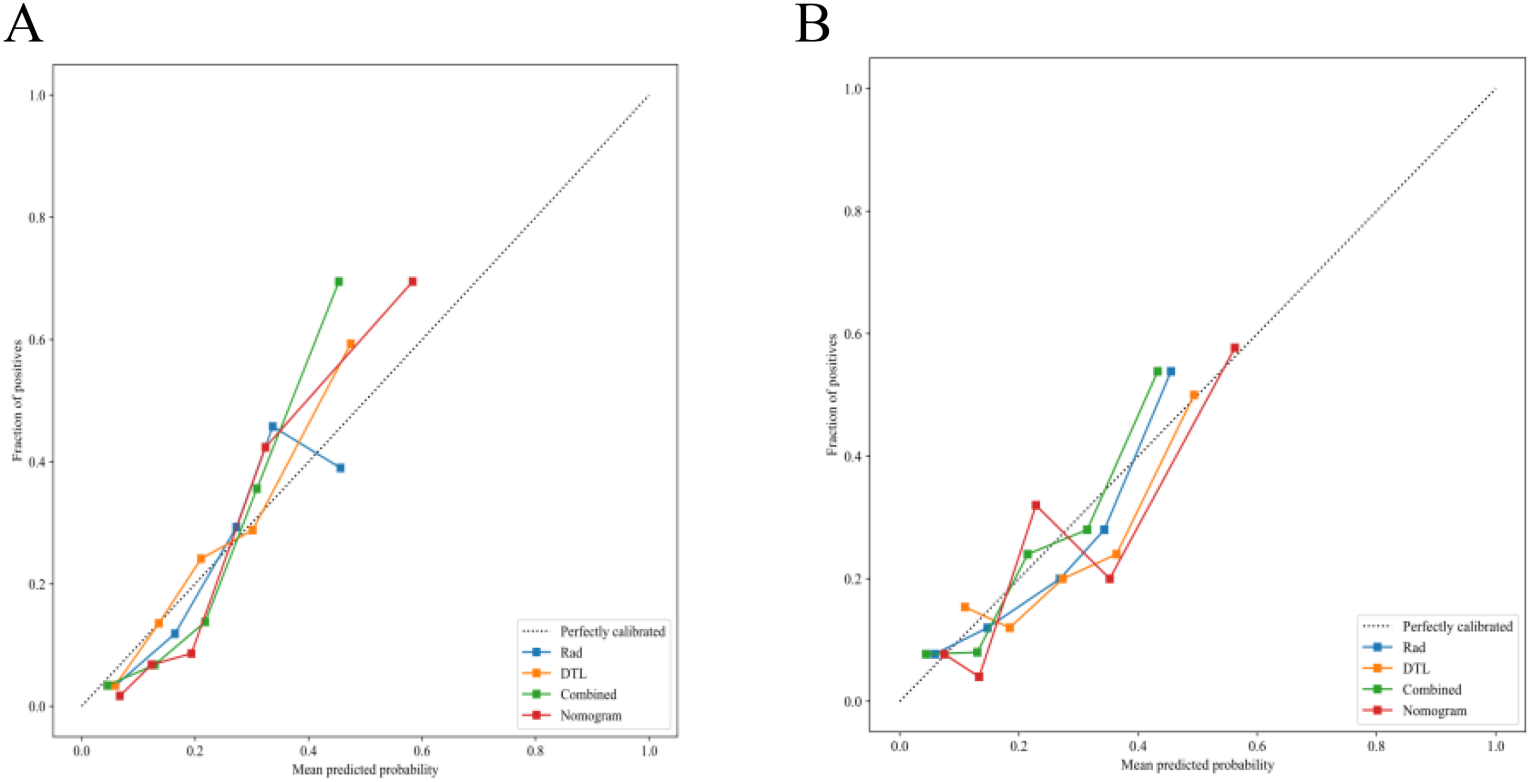
Calibration performance of the nomogram. **(A)** Training set calibration curve demonstrating concordance between the predicted and observed pelvic lymph node metastasis rates (Hosmer-Lemeshow test: *p* = 0.072). **(B)** Testing set calibration curve validating model generalizability, with maintained agreement between predictions and outcomes (Hosmer-Lemeshow test: *p* = 0.131).

**Figure 15:**
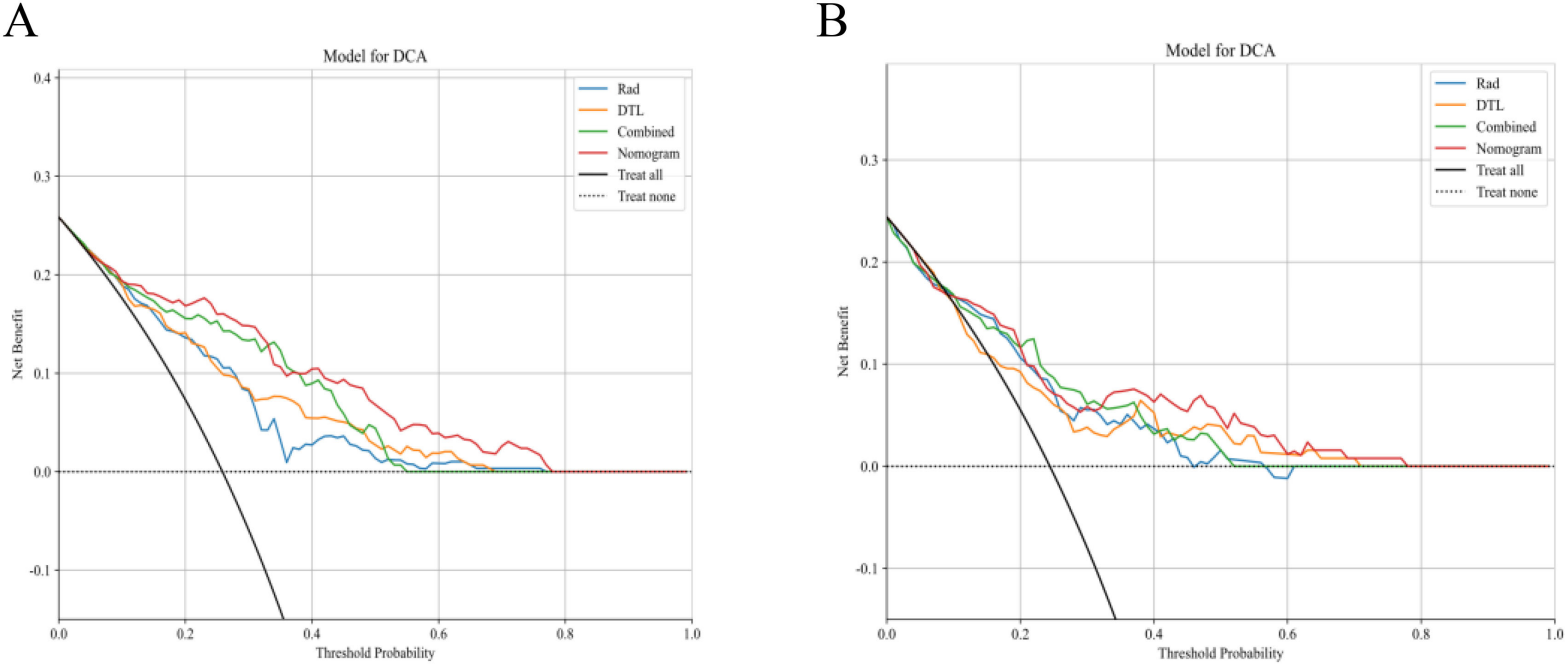
Decision curve analysis of the clinical utility of the model. **(A)** Training set: the nomogram achieves significantly higher net benefit than the radiomics, DTL, and fusion models across clinically relevant threshold probabilities (10%–50%). **(B)** Testing set: sustained superiority of the nomogram in the validation cohort, with net benefit outperforming that of the comparator models at all decision thresholds. DTL, deep transfer learning.

The fusion model was combined with independent clinical predictors (maximum tumor diameter and RBC count) to develop a nomogram (**Figure 12**). The nomogram exhibited superior discrimination, with training and testing AUCs of 0.871 (95% CI: 0.8274–0.9156) and 0.764 (95% CI: 0.6604–0.8678), respectively, outperforming the radiomics, DTL, and fusion models (**Table 6**, **Figure 13**). Calibration curves demonstrated excellent agreement between the predicted and observed PLNM probabilities in both cohorts (Hosmer-Lemeshow test: training, *p* = 0.072; testing, *p* = 0.131; **Figure 14**). Decision curve analysis confirmed that the nomogram provided a greater net benefit across clinically relevant threshold probabilities (10%–50%) than that associated with alternative models (**Figure 15**).

**Table 6:** Comparison of AUCs of radiomics, DTL, fusion, and nomogram models.

Model	AUC	95% CI	Accuracy	Sensitivity	Specificity	PPV	NPV
Radiomics-Training	0.749	0.6906–0.8071	0.653	0.829	0.592	0.414	0.908
DTL-Training	0.782	0.7231–0.8400	0.694	0.776	0.665	0.447	0.895
Fusion-Training	0.857	0.8086–0.9064	0.762	0.829	0.739	0.525	0.925
Nomogram-Training	0.871	0.8274–0.9156	0.789	0.882	0.757	0.558	0.948
Radiomics-Testing	0.729	0.6249–0.8334	0.677	0.677	0.677	0.404	0.867
DTL-Testing	0.702	0.5853–0.8193	0.772	0.548	0.844	0.531	0.853
Fusion-Testing	0.753	0.6494–0.8560	0.669	0.806	0.625	0.410	0.909
Nomogram-Testing	0.764	0.6604–0.8678	0.780	0.581	0.844	0.545	0.862

AUC, area under the curve; DTL, deep transfer learning; CI, confidence interval; PPV, positive predictive value; NPV, negative predictive value; Fusion, radiomics-DTL fusion model.

## Discussion

### Radiomics and deep learning synergy​

While the acquisition of ultrasound images may be subject to operator variability,

quantitative characterization of tumor heterogeneity through radiomic feature extraction enables the objective assessment of metastatic propensity, thereby circumventing subjective variability in image interpretation (25). In our study, we implemented several strategies to enhance robustness against operator variability. First, all sonographers followed a standardized imaging protocol to minimize acquisition differences. More importantly, during feature extraction, we performed a rigorous reproducibility assessment based on both inter- and intra-observer intraclass correlation coefficients (ICCs). Features with ICCs below 0.75 were excluded, ensuring that only stable and reproducible features were used for model construction. This process helps to filter out features that are highly sensitive to segmentation differences or acquisition parameters, thereby increasing the generalizability and reliability of the radiomic signature (26, 27). Future studies employing automated segmentation algorithms could further reduce this potential source of variability. Earlier research has demonstrated that radiomics-based approaches can effectively predict lymph node metastasis across various cancer types (25, 28, 29). The radiomic component of our model demonstrated robust validation performance (testing AUC = 0.729), which was consistent with the performance of MRI-based radiomic prediction of nodal involvement (AUC = 0.83) in a meta-analysis of cervical cancer patients (9). This concordance suggests the potential of ultrasound-based radiomics as a cost-effective alternative to advanced imaging modalities, particularly in resource-constrained settings.

DL, a subset of machine learning, enables computational models featuring multiple processing layers to acquire hierarchical representations of data across various levels of abstraction (30). In numerous real-world scenarios, convolutional neural networks that have been initially trained on the ImageNet dataset are widely utilized, a technique commonly referred to as transfer learning (31, 32). In our study, DTL architectures exhibited paradoxical performance characteristics, with superior training accuracy (AUC = 0.782) versus diminished validation metrics (AUC = 0.702). This performance gap aligns with findings in gastrointestinal oncology research, where DTL models consistently exhibit a higher risk of overfitting than radiomic approaches (33, 34). In our study, the DTL model was inferior to the conventional radiomic model in the testing set. The possible reasons for this are as follows: First, DTL requires a large number of training sets, whereas this is a single-center study; a large multi-center is needed for further training in the future. Second, despite the strong performance of DTL in diverse classification and prediction tasks, its inherent lack of interpretability, characteristic of black-box algorithms, limits its broader applicability (35, 36). Future iterations incorporating three-dimensional ROI reconstructions and attention mechanisms may enhance the generalizability of DTL (37).

### Advantages of multimodal fusion​

The feature-level integration of radiomic and DTL biomarkers generated synergistic diagnostic improvements, with the testing AUC (0.753) of the fusion model exceeding those of standalone approaches. This aligns with the findings of Wang et al., who found that the fusion of radiomics and deep learning features achieved a testing AUC of 0.934 for differentiating benign and malignant parotid gland tumors, significantly surpassing standalone radiomics (AUC = 0.853) and deep learning models (AUC = 0.883) (16). The fusion model demonstrated enhanced capability in detecting small metastatic foci (<5 mm) by leveraging complementary features: radiomics quantified margin irregularities (e.g., GLRLM_ShortRunEmphasis), while DTL identified subtle perilesional vascular patterns. This multimodal synergy directly addresses the longstanding limitation of conventional imaging in identifying micrometastases—a challenge highlighted in studies such as that by Liu et al. (2017), who found that conventional CT criteria (e.g., size thresholds) struggled to detect sub-centimeter nodal metastases in esophageal cancer (38). By integrating heterogeneous biomarkers, the fusion approach overcomes the sensitivity-precision trade-off inherent to single-modality methods, aligning with advancements in radiomics-deep learning fusion frameworks observed in other oncologic contexts (17, 38).

### Toward a multiparametric ultrasound fusion model

Our study utilized conventional B-mode ultrasound images, which are the most widely available and cost-effective. However, we acknowledge that emerging ultrasound technologies such as three-dimensional (3D) ultrasonography can provide additional quantitative information on tumor volume. Volumetric data from 3D imaging captures the full spatial complexity of a tumor (39). SWE offers unique insights into underlying biological characteristics. Tissue stiffness, as measured by SWE, often correlates with pathological processes such as fibrosis and cellular proliferation, which are hallmarks of malignancy (19, 40, 41). Contrast-enhanced ultrasonography can assess the vitality of tumors through contrast enhancement, and can depict the real-time dynamic perfusion of tumors (42). These parameters have shown promise in characterizing tumor aggressiveness and predicting lymph node metastasis. Integrating these multi-parametric ultrasound features into our fusion model represents a compelling direction for future research, potentially further boosting predictive accuracy.

### Clinical parameter integration​

Multivariate analysis identified maximum tumor diameter (OR = 1.055) and RBC count (OR = 0.646) as independent predictors of PLNM, corroborating established oncopathological mechanisms. Numerous research studies have indicated that tumor size serves as a standalone predictor for lymph node metastasis in cervical cancer; tumor size directly correlates with the probability of lymphatic invasion (43, 44). Thus, large tumors (tumor diameter > 4 cm) are a risk factor for nodal metastasis and considerably increase the incidence of PLNM (45). Conversely, anemia (reflected by a low RBC count) can be associated with chronic tumor hemorrhage and inflammatory microenvironment modifications that are conducive to metastatic spread (46). Considering the influence of clinical factors on PLNM, we developed a nomogram incorporating the radiomics signature, the DTL signature, maximum tumor diameter, and RBC count. The nomogram displayed good calibration and excellent performance to evaluate PLNM status with an AUC of 0.871 in the training cohort and 0.764 in the test cohort. The incorporation of these clinical parameters with imaging biomarkers in the nomogram created a biologically plausible decision tool that outperformed single-modal models, which is consistent with the results reported in the literature (47, 48). However, it is important to note that our model was developed and validated on a single-center retrospective dataset. While internal validation showed promising results, the generalizability of our model needs to be confirmed in multi-center, prospective studies with diverse patient populations and imaging protocols.

### Technical and clinical implications​

This investigation represents the first implementation of ultrasound-based radiomics-DTL fusion for cervical cancer nodal staging, addressing 2 critical clinical needs (1): cost-effective alternatives to MRI and PET-CT in low-resource settings (4, 10), and (2) quantitative standardization of subjective ultrasound interpretation (25). The achieved diagnostic accuracy (training AUC: 0.871) positions our nomogram competitively against MRI-based models requiring specialized sequences and contrast administration (10, 49). Furthermore, the compatibility of our methodology with portable ultrasound systems enables potential deployment in screening/telemedicine contexts, which is particularly valuable in geographically dispersed populations. However, a performance gap was observed between the training (AUC = 0.871) and testing (AUC = 0.764) cohorts. This is an expected phenomenon in machine learning, reflecting the model’s adaptation to the specific patterns of the training data. It is crucial to highlight that our modeling pipeline incorporated several strategies to mitigate overfitting, including LASSO regularization and 10-fold cross-validation during feature selection. The testing AUC of 0.764, therefore, represents a more realistic estimate of the model’s performance on unseen data, which remains clinically valuable and competitive with existing literature (50). Most importantly, the DCA demonstrated that the nomogram provided superior clinical net benefit across a wide range of threshold probabilities (10%–50%) in the testing set, underscoring its potential utility in clinical decision-making despite the observed drop in AUC.

### Limitations and future directions​

Four principal limitations warrant consideration. First, the most significant limitation of this study is its single-center, retrospective nature. The absence of an external validation cohort from a different institution limits the assessment of the model’s generalizability and robustness against variations in ultrasound equipment and operator expertise. Second, two-dimensional ROI analysis disregards volumetric heterogeneity patterns, which are increasingly being recognized as metastatic predictors. Third, the exclusion of functional ultrasound parameters (Doppler indices, elastography) omitted potentially discriminative hemodynamic data. Fourth, the monocentric training dataset (n = 294) may insufficiently represent global population diversity, requiring multicenter expansion for clinical implementation.

In the future, we will prioritize external validation in a multi-center setting to confirm the clinical translatability of our nomogram, and prospective validation efforts should focus on advancing multidimensional tumor assessment by integrating volumetric three-dimensional sampling techniques with dynamic perfusion monitoring across treatment timelines, while concurrently establishing molecular validation frameworks through longitudinal tracking of circulating tumor DNA biomarkers.

## Conclusions

The nomogram developed in this study establishes a clinically viable framework for preoperative PLNM prediction in patients with cervical cancer, and synergistically integrates the accessibility of ultrasonography with advanced computational analytics. By achieving a diagnostic accuracy comparable to those of resource-intensive modalities through multimodal feature fusion, this approach holds particular promise for optimizing therapeutic stratification in resource-variable healthcare ecosystems.

## Data Availability

The original contributions presented in the study are included in the article/**Supplementary Material**. Further inquiries can be directed to the corresponding author/s.
