# Supplementary figures and images for: Fusion model combining ultrasound-based radiomics and deep transfer learning with clinical parameters for preoperative prediction of pelvic lymph node metastasis in cervical cancer
Source: Front Oncol. 2025 Nov 13;15:1681029. doi: 10.3389/fonc.2025.1681029 (PMC12658780; doi:10.3389/fonc.2025.1681029)

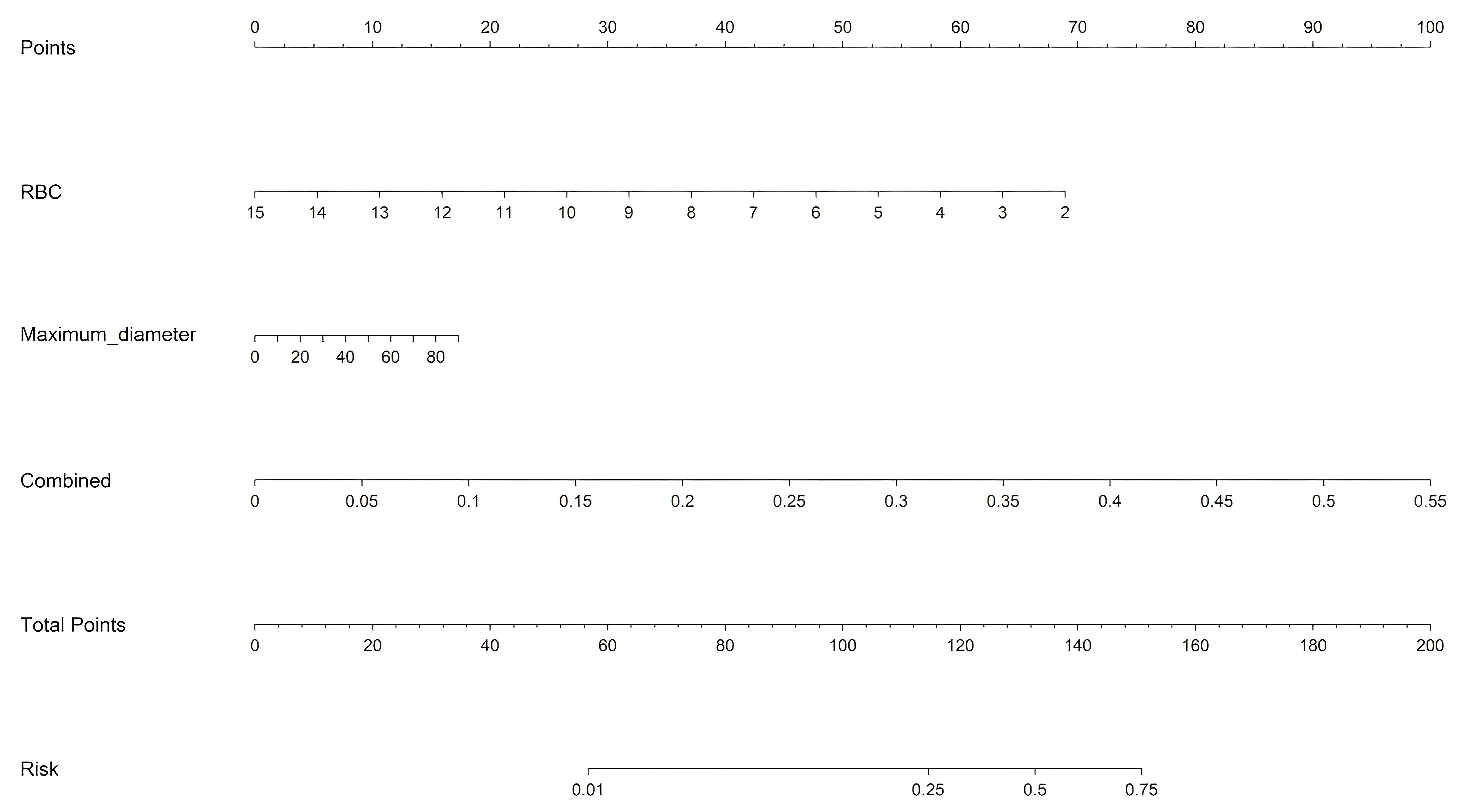

Supplement: Supplementary file 1 [file Image1.jpeg]

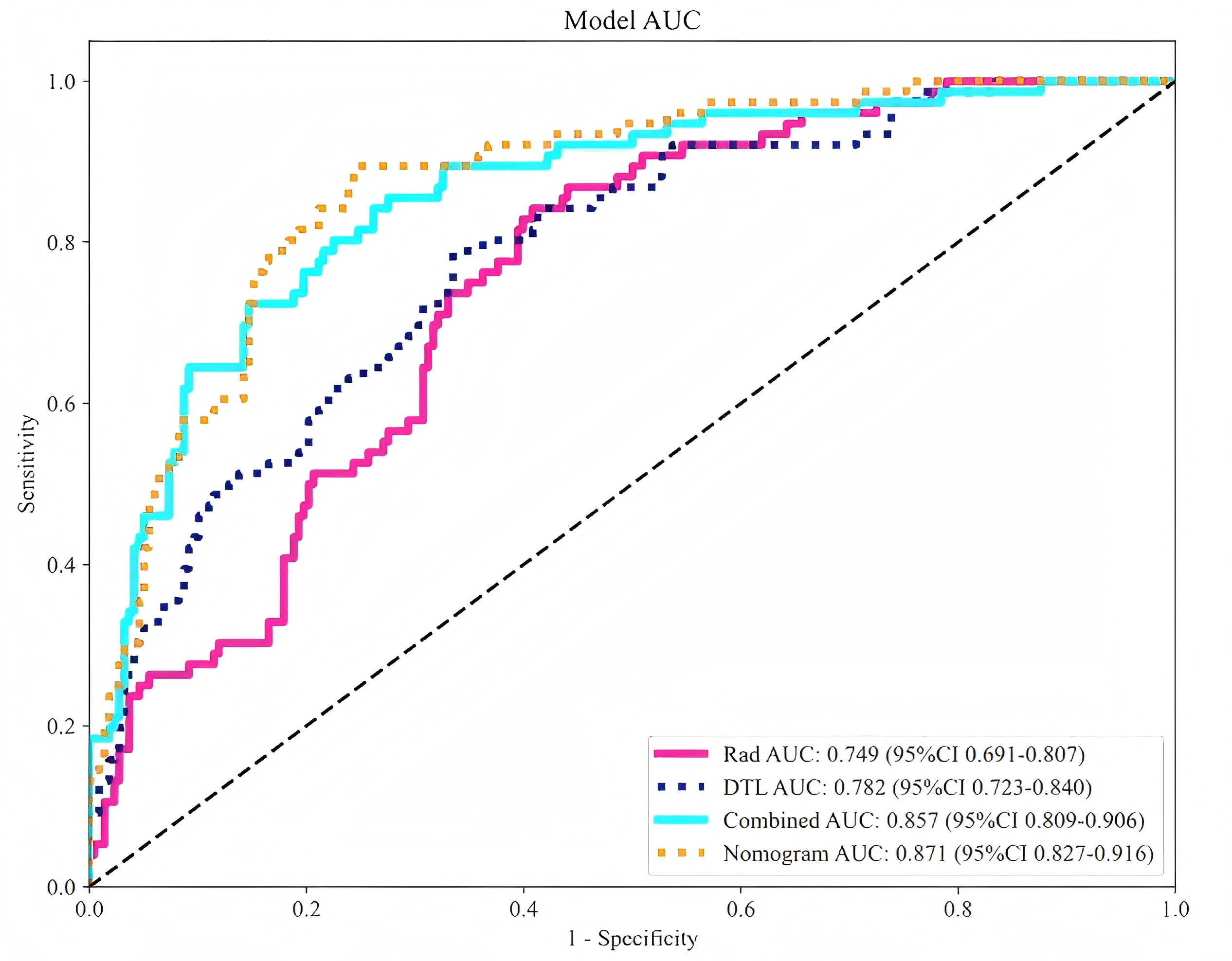

Supplement: Supplementary file 2 [file Image2.jpeg]

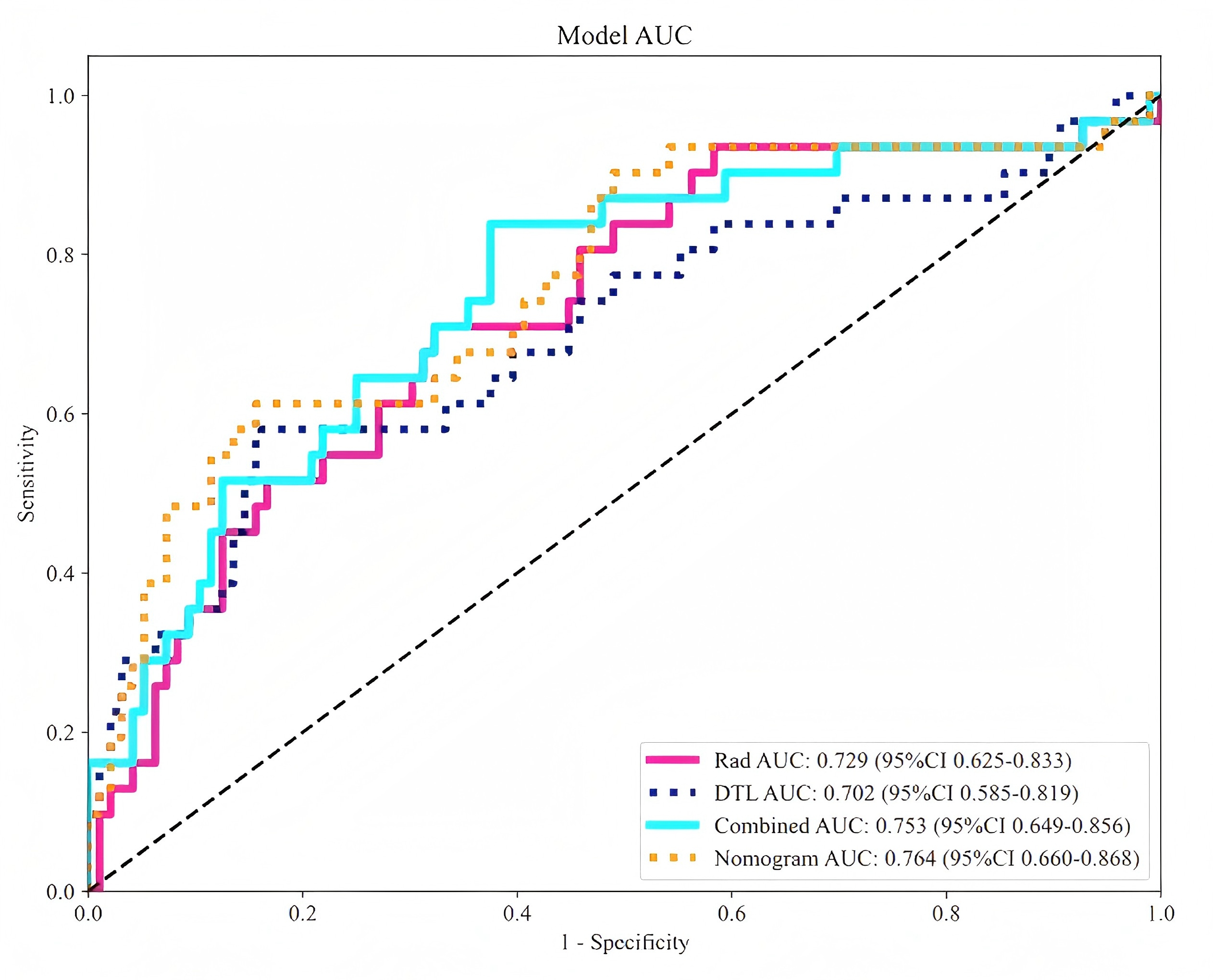

Supplement: Supplementary file 3 [file Image3.jpeg]

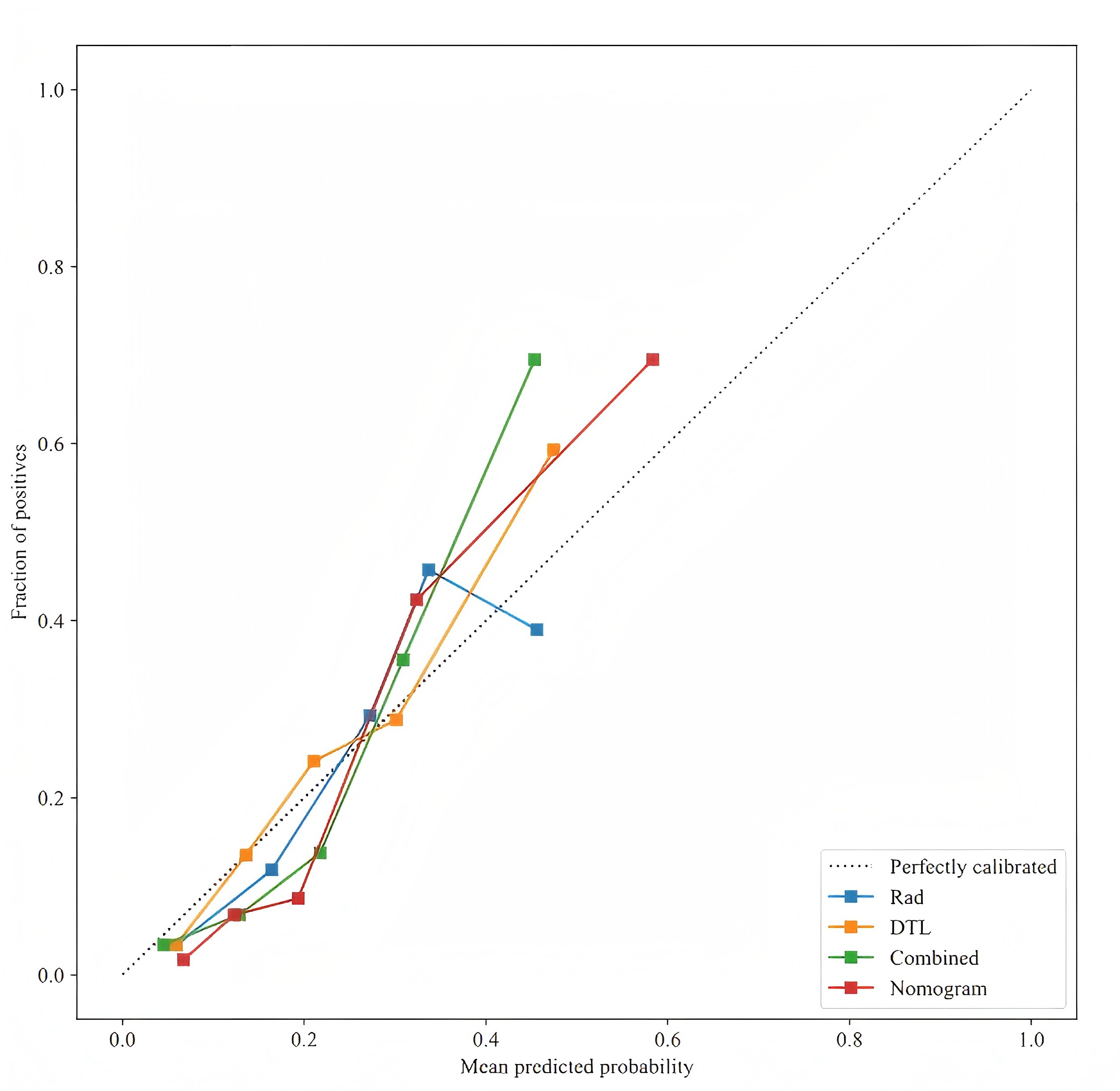

Supplement: Supplementary file 4 [file Image4.jpeg]

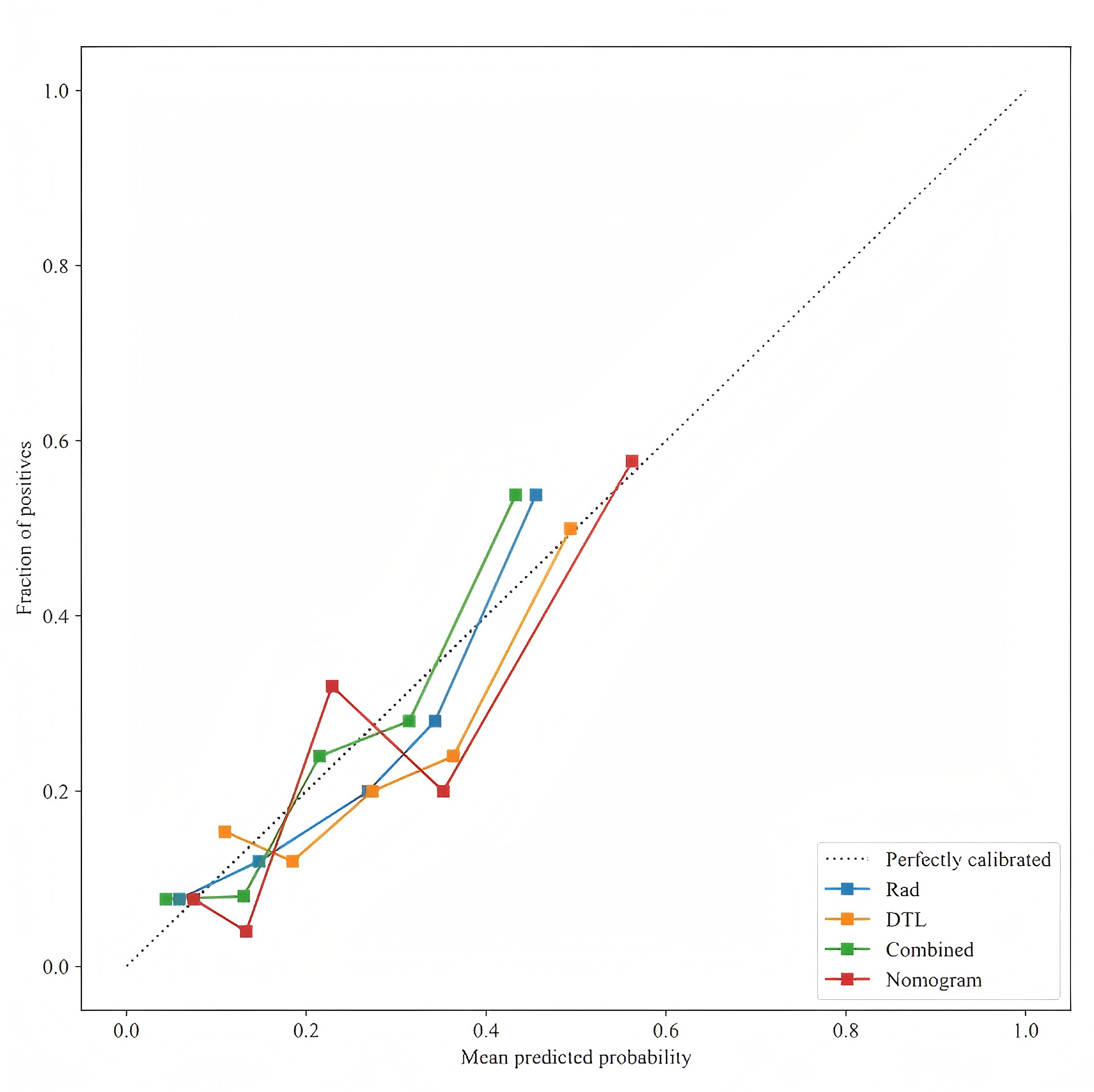

Supplement: Supplementary file 5 [file Image5.jpeg]

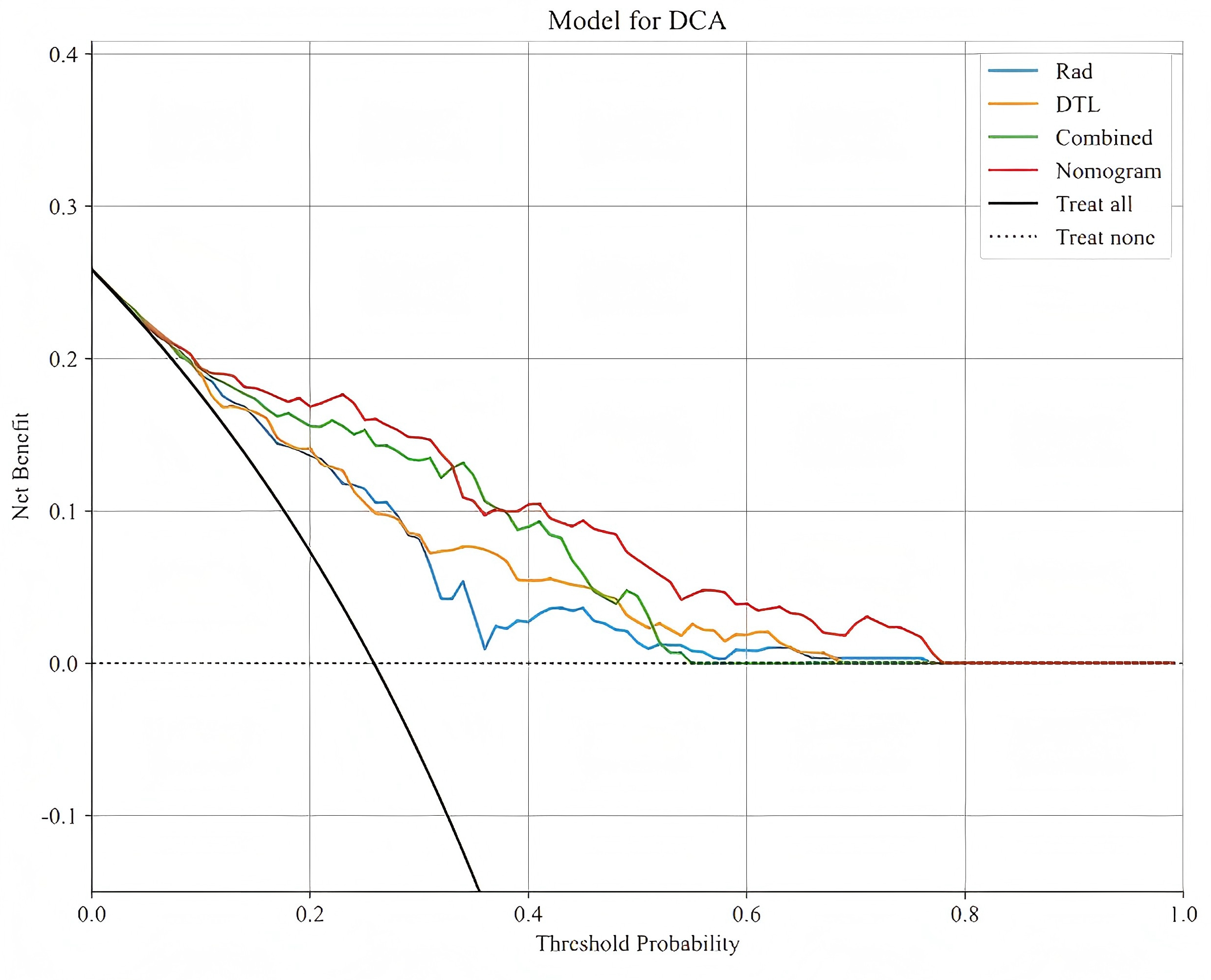

Supplement: Supplementary file 6 [file Image6.jpeg]

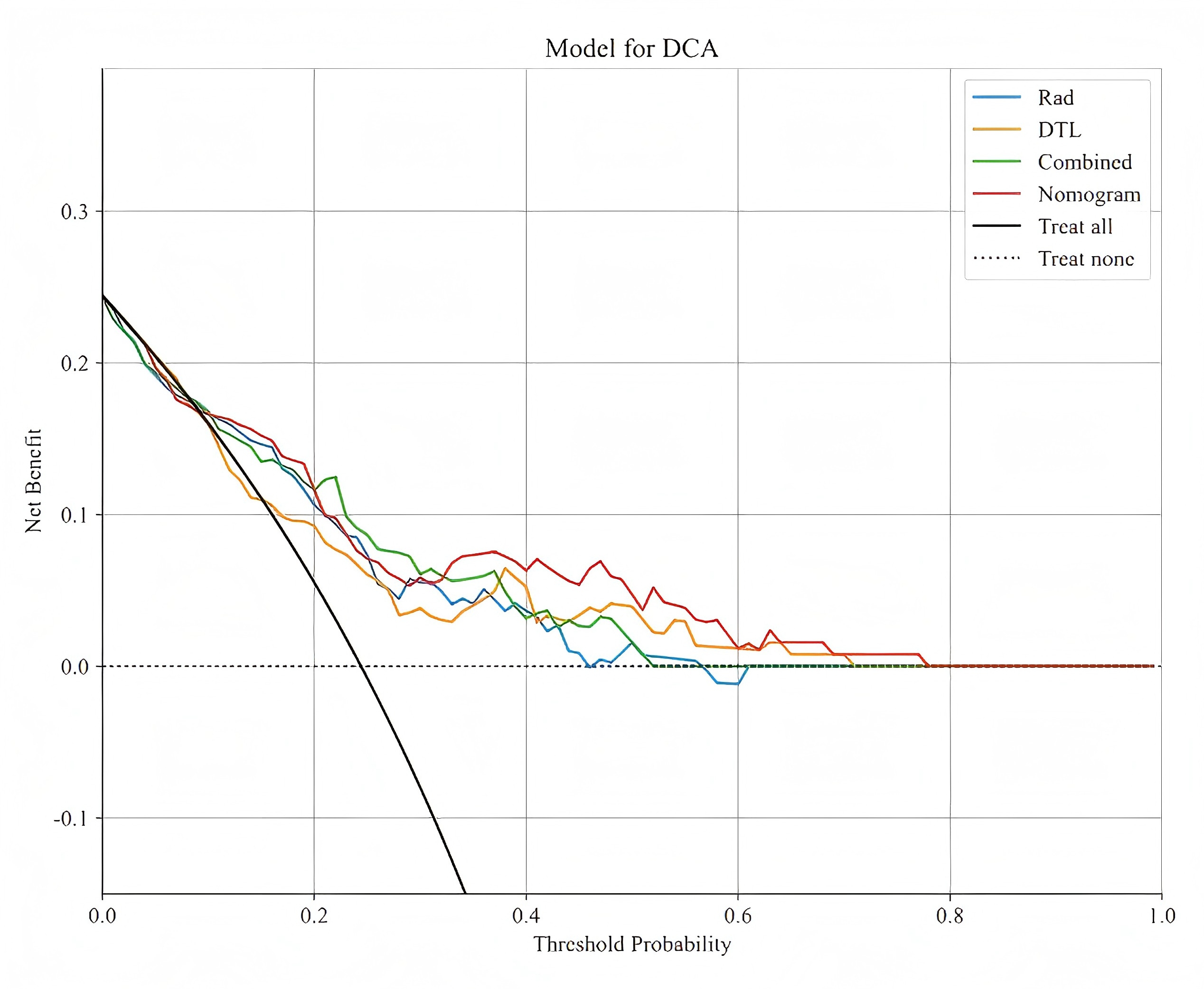

Supplement: Supplementary file 7 [file Image7.jpeg]
